# Storage Space Allocation Strategy for Digital Data with Message Importance

**DOI:** 10.3390/e22050591

**Published:** 2020-05-25

**Authors:** Shanyun Liu, Rui She, Zheqi Zhu, Pingyi Fan

**Affiliations:** 1 Department of Electronic Engineering, Tsinghua University, Beijing 100084, China; liushany16@mails.tsinghua.edu.cn (S.L.); sher15@mails.tsinghua.edu.cn (R.S.); zhuzq18@mails.tsinghua.edu.cn (Z.Z.); 2 Beijing National Research Center for Information Science and Technology (BNRist), Beijing 100084, China

**Keywords:** lossy compression storage, optimal allocation strategy, weighted reconstruction error, message importance measure, importance coefficient

## Abstract

This paper mainly focuses on the problem of lossy compression storage based on the data value that represents the subjective assessment of users when the storage size is still not enough after the conventional lossless data compression. To this end, we transform this problem to an optimization, which pursues the least importance-weighted reconstruction error in data reconstruction within limited total storage size, where the importance is adopted to characterize the data value from the viewpoint of users. Based on it, this paper puts forward an optimal allocation strategy in the storage of digital data by the exponential distortion measurement, which can make rational use of all the storage space. In fact, the theoretical results show that it is a kind of restrictive water-filling. It also characterizes the trade-off between the relative weighted reconstruction error and the available storage size. Consequently, if a relatively small part of total data value is allowed to lose, this strategy will improve the performance of data compression. Furthermore, this paper also presents that both the users’ preferences and the special characteristics of data distribution can trigger the small-probability event scenarios where only a fraction of data can cover the vast majority of users’ interests. Whether it is for one of the reasons above, the data with highly clustered message importance is beneficial to compression storage. In contrast, from the perspective of optimal storage space allocation based on data value, the data with a uniform information distribution is incompressible, which is consistent with that in the information theory.

## 1. Introduction

As large amounts of mobile devices such as Internet of things (IoT) devices or smartphones are utilized, the contradiction between limited storage space and sharply increasing data deluge becomes increasingly serious in the era of big data [1,2]. This exceedingly massive data makes the conventional data storage mechanisms inadequate within a tolerable time, and therefore the data storage is one of the major challenges in big data [3]. Note that storing all the data becomes more and more dispensable nowadays, and it is also not conducive to reduce data transmission costs [4,5]. In fact, data compression storage is widely adopted in many applications, such as IoT [2], industrial data platform [6], bioinformatics [7], wireless networking [8]. Thus, the research on data compression storage becomes increasingly paramount and compelling nowadays.

In conventional source coding, data compression is carried out by removing the data redundancy, where short descriptions are assigned to the most frequent class [9]. Based on it, the tight bounds for lossless data compression are given. In order to further increase the compression rate, one needs to use more information. A quintessential example is to use some side information [10]. Another possible solution is to compress the data with quite a few losses first and then reconstruct them with acceptable distortion, which is referred to as lossy compression [11,12,13]. Some adaptive compressions are adopted extensively. For example, Reference [14] proposed an adaptive compression scheme in IoT systems, and Reference [15] investigated the backlog-adaptive source coding system in terms of age of information. In fact, most of the previous compression methods usually carried out compression by means of contextual data or leveraging data transformation techniques [4].

Although these previous methods of data compression perform satisfactorily in their respective application scenarios, there is still much room for improvement when facing rapidly growing large-scale data. Moreover, they also do not take the data value into account. This paper focuses on the problem of how to further compress data with acceptable distortion to implement the specified requirements in data storage when the storage size is still not enough to guarantee the lossless storage after the conventional lossless data compression. This paper will realize this goal by reallocating storage space based on the data value which represents the subjective assessment of users. Here, we take the importance-aware weighting in the weighted reconstruction error to measure the total cost in data storage with unequal costs.

Generally, users prefer to care about the crucial part of data that attracts their attention rather than the whole data itself. In many real-world applications, such as cost-sensitive learning [16,17,18] and unequal error protection [19,20], different errors bring different costs. To be specific, the distortion in the data that users care about may be catastrophic if the loss of some data being insignificant for users is allowed. Similar to coresets [21], the data needing to be processed was reduced to those users as the main focus rather than the whole data set. Unlike coresets, the data needing to be processed in this paper no longer pursues approximately representing the raw data, and it is expected to minimize the storage cost with respect to the importance weighting value. In fact, although the data deluge sharply increases, the significant data that users care about is still rare in a lot of scenarios of big data. In this sense, it can be regarded as the sparse representation from the perspective of the data value, and we can use it to compress data.

Alternatively, it is interesting to achieve data compression by storing a fraction of data, which preserves as much information as possible regarding the data that users care about [22,23]. This paper also employs this strategy. However, there are subtle but critical differences between the compression storage strategy proposed in this paper with those in Reference [22,23]. In fact, Reference [22] focused on Pareto-optimal data compression, which presents the trade-off between retained entropy and class information. However, this paper puts forward an optimal compression storage strategy for digital data from the viewpoint of message importance, and it gives the trade-off between the relative weighted reconstruction error (RWRE) and the available storage size. Furthermore, the compression method based on message importance was preliminarily discussed in Reference [23] to solve the big data storage problem in wireless communications, while this paper will aim to discuss the optimal storage space allocation strategy with limited storage space, in general, based on message importance. Moreover, the constraints are also different. That is, the available storage size is limited in this paper, while the total code length of all the events is given in Reference [23].

From users’ attention viewpoint, the data value can be considered as the subjective assessment of users on the importance of data. Actually, much of the research in the last decade suggested that the study from the perspective of message importance is rewarding to obtain new findings [20,24,25]. Thus, there may be effective performance improvement in storage systems when taking message importance into account. For example, Reference [26] discussed the lossy image compression method with the aid of a content-weighted importance map. Since any quantity can be seen as important if it agrees with the intuitive characterization of the user’s subjective degree of concern of data, the cost in data reconstruction for specific user preferences is regarded as the importance in this paper, which will be used as the weight in the weighted reconstruction error.

Since we desire to achieve data compression by keeping only a small portion of important data and abandoning less important data, this paper mainly focuses on the case where only a fraction of data take up the vast majority of the users’ interests. Actually, these scenarios are not rare in big data. A quintessential example should be cited that the minority subset detection is overwhelmingly paramount in intrusion detection [27,28]. Moreover, this phenomenon is also exceedingly typical in financial crime detection systems for the fact that only a few illicit identities catch our eyes to prevent financial frauds [29]. Actually, when a certain degree of information loss can be acceptable, people prefer to take high-probability events for granted and abandon them to maximize the compressibility. These cases are referred to as *small-probability event scenarios* in this paper. In order to depict the message importance in small-probability event scenarios, message importance measure (MIM) was proposed in Reference [30]. Furthermore, MIM is fairly effective in many applications of big data, such as IoT [31], mobile edge computing [32]. In addition, Reference [33] expanded MIM to the general case, and it presented that MIM can be adopted as a special weight in designing the recommendation system. Since there is no universal data value model, we might as well take the case where the MIM describes the cost of the error as a quintessential example to analyze the property of the optimal storage space allocation strategy.

In this paper, we firstly propose a particular storage space allocation strategy for digital data on the best effort in minimizing the importance-weighted reconstruction error when the total available storage size is provided. For digital data, we formulate this problem as an optimization problem, and present the optimal storage strategy by means of a kind of restrictive water-filling. For the given available storage size, the storage size is mainly determined by the values of message importance and probability distribution of event class in a data sequence. In fact, this optimal allocation strategy adaptively prefers to provide more storage size for crucial data classes in order to make the rational use of resources, which is in accord with the cognitive mechanism of human beings.

Afterward, we focus on the properties of this optimal storage space allocation strategy when the importance weights are characterized by MIM. It is noted that there is a trade-off between the RWRE and the available storage size. The constraints on the performance of this storage system are true, and they depend on the importance coefficient and the probability distribution of events classes. On the one hand, the RWRE increases with the increasing of the absolute value of importance coefficient for the fact that the overwhelming majority of important information will gather in a fraction of data as the importance coefficient increases to negative/positive infinity, which suggests the influence of users’ preferences. On the other hand, the compression performance is also affected by probability distribution of event classes. In fact, the more closely the probability distribution matches the requirement of the small-probability event scenarios, the more effective this compression strategy becomes. Furthermore, it is also obtained that the RWRE in a uniform distribution is larger than any other distributions for the same available storage size. In this regard, the uniform distribution is incompressible from the perspective of optimal storage space allocation based on data value, which is consistent with the conclusion in information theory [34].

The main contributions of this paper can be summarized as follows. (1) It proposes a new digital data compression strategy taking message importance into account, which can help improve the design of a big data storage system. (2) We illuminate the properties of this new method, which can characterize the trade-off between the RWRE and the available storage size. (3) It shows that the data with highly clustered message importance is beneficial to compression storage, and it also finds that the data with a uniform information distribution is incompressible from the perspective of optimal storage space allocation based on data value, which is consistent with that in information theory.

The rest of this paper is organized as follows. The system model is introduced in Section 2, including the definition of weighted reconstruction error, distortion measure, and problem formulation. In Section 3, we solve the problem of optimal storage space allocation in three kinds of system models and give the solutions. The properties of this optimal storage space allocation strategy based on MIM are fully discussed in Section 4. The effects of the importance coefficient and the probability of event classes on RWRE are also investigated in detail. Section 5 illuminates the properties of this optimal storage strategy when the importance weight is characterized by Non-parametric MIM. The numerical results are shown and discussed in Section 6, which verifies the validity of the developed theoretical results in this paper. Finally, we give the conclusion in Section 7.

## 2. System Model

This section introduces the system model, including the definition of the weighted reconstruction error, the modeling of distortion measure, in order to illustrate how we formulate the lossy compression problem as an optimization problem for digital data based on message importance. In order to make the formulation and discussion more clear, the main notations in this paper are listed in Table 1.

### 2.1. Modeling Weighted Reconstruction Error Based on Message Importance

The data storage system may lack storage space frequently when facing a super-large scale of data to store. When the storage size is still not enough after the lossless conventional data compression, the optimum allocation of storage space based on data value may be imperative. For this purpose, we consider the following storage system, which stores *K* pieces of data. Let $x=x_{1},x_{2},\ldots ,x_{k},\ldots ,x_{K}$ be the sequence of raw data. Assume that all the data redundancy have been removed after the lossless conventional data compression, and each data $x_{k}$ needs to take up storage space with size of $Sx_{k}$ if this data can be recovered without any distortion. However, in many scenarios of big data, the storage size is still not enough in this case. That is to say, the actual required storage space $\sum_{k=1}^{K}Sx_{k}$ is larger than the maximum available storage space TK, where *T* is the maximum available average storage size.

In fact, users prefer to care about the paramount part of data that attracts their attention rather than the whole data itself. In this perspective, storing all data without distortion may be unnecessary. Considering that the natural distribution of storage space is not invariably reasonable and the high value data in big data is usually sparse, the rational storage space allocation by minimizing the loss of data value may solve the above problem of insufficient storage space, if a certain amount of data value is allowed to be lost. After the data compression by means of the rational storage space allocation, we use ${\hat{x}}_{1},{\hat{x}}_{2},\ldots ,{\hat{x}}_{k},\ldots ,{\hat{x}}_{K}$ to denote the compressed data sequence, and assume that the compressed data ${\hat{x}}_{k}$ takes up storage space with size of $S{\hat{x}}_{k}$ in practice for 1≤k≤K.

The lossy data compression usually pursues the least the storage cost while retaining as much information users required as possible [22]. In the lossless conventional data compression, the costs of different data are assumed to be the same. However, different kinds of errors may result in unequal costs in many real-world applications [16,17,18,19]. In this model, we use the notation $W_{k}$ to denote the error cost for the reconstructed data. Namely, $W_{k}$ is with respect to the data value of data $x_{k}$, and it is regarded as the message importance in this paper. Here, we define the weighted reconstruction error to describe the total cost in data storage with unequal costs, which is given by

(1) $$\begin{array}{c} D\left(x,W\right)=\frac{1}{K}\sum_{k=1}^{K}W_{k}D_{f}\left(Sx_{k},S{\hat{x}}_{k}\right), \end{array}$$

where $D_{f}\left(Sx_{k},S{\hat{x}}_{k}\right)$ characterizes the distortion between the raw data and the compressed data in data reconstruction, which characterizes the loss degree of data value with allocated storage size.

**Figure 1:**
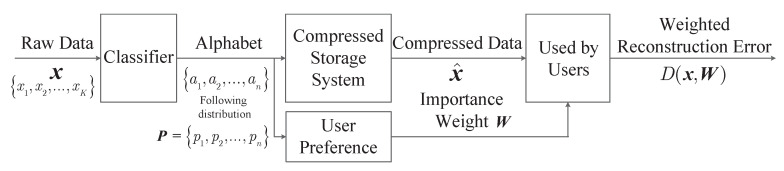
Pictorial representation of the system model.

Consider the situation where the data is stored according to its category for easier retrieval, which can also make the recommendation system based on it more effective [33]. Since data classification is becoming increasingly convenient and accurate nowadays due to the rapid development of machine learning [35,36], this paper assumes that the event class can be easily detected and known in the storage system. Moreover, assume the data that belongs to the same class has the same importance-weight and occupies the same storage size. Hence, x can be seen as a sequence of *K* symbols from an alphabet $\left\{a_{1},a_{2},\ldots ,a_{n}\right\}$ where $a_{i}$ represents event class *i*. This storage model is summarized and shown in Figure 1. In this case, the weighted reconstruction error based on importance is formulated as

(2) $$\begin{array}{cc} D(x,W) & =\sum_{i=1}^{n}\frac{N(a_{i}|x)}{K}W_{i}D_{f}\left(Sa_{i},S{\hat{a}}_{i}\right) \end{array}$$

(2a) $$\begin{array}{c} =\sum_{i=1}^{n}p_{i}W_{i}D_{f}\left(Sa_{i},S{\hat{a}}_{i}\right), \end{array}$$

where $N(a_{i}|x)$ is the number of times the *i*-class occurs in the sequence x. Let $p_{i}=N(a_{i}|x)/K$ denote the probability of event class *i* in data sequence x.

### 2.2. Modeling Distortion between the Raw Data and the Compressed Data

We focus on the formula of $D_{f}$ in this part, which characterizes the distortion between the raw data and the compressed data with specified storage size. Usually, there is no universal characterization of distortion measure, especially in speech coding and image coding [34]. In fact, $D_{f}$ should characterize the loss degree of data value with allocated storage size. In this respect, the conventional distortion measures are not appropriate since they do not take unequal costs into account. In order to facilitate the analysis and design, this paper proposes an exponential distortion measure to discuss the following special case.

We assume that the data is digital and ignore the storage formats and standards in concrete application environments. On its application fields, it may be useful in some scenarios with counting systems, such as finance, or medicine, as the general merchandise. Let the description of the raw data $a_{i}$ be $L_{i}$ bits, and $a_{i}=\sum_{j=0}^{L_{i}-1}b_{j}\times r^{j}$ where *r* is radix (r>1). Actually, the radix represents the base of the system in practical application, such as r=2 in a binary system. In particular, $L_{i}$ will approach the infinite number if $a_{i}$ is an arbitrary real number. When the storage size is still not enough after the lossless conventional data compression, there is only $l_{i}$ bits assigned to it in order to compress data further based on the message importance. For convenience, the smaller $\left(L_{i}-l_{i}\right)$ numbers are discarded in this process. When restoring the compressed data, the discarded digits are set to the same pre-specified number or random numbers in the actual system. Let $b_{j}^{*}$ be the (j+1)-th discarded digit for $j=0,1,\cdots ,L_{i}-l_{i}-1$, and assume that $b_{j}^{*}$ is a random number in {0,…,r−1}. In this case, the compressed data is ${\hat{a}}_{i}=\sum_{j=L_{i}-l_{i}}^{L_{i}-1}b_{j}\times r^{j}+\sum_{j=0}^{L_{i}-l_{i}-1}b_{j}^{*}\times r^{j}$. As a result, the absolute error is $|a_{i}-{\hat{a}}_{i}|$, which meets

(3) $$\begin{array}{c} \left|a_{i}-{\hat{a}}_{i}\right|=\left|\sum_{j=0}^{L_{i}-l_{i}-1}\left(b_{j}-b_{j}^{*}\right)\times r^{j}\right|\leq r^{L_{i}-l_{i}}-1. \end{array}$$

When $l_{i}=0$, which means there is no information stored, the supremum of absolute error reaches the maximum and it is $|a_{i}-{\hat{a}}_{i}|\leq r^{L_{i}}-1$. In order to better weigh the different costs, we define the relative error by normalizing the absolute error to the interval [0,1] based on the above maximum absolute error $r^{L_{i}}-1$. Moreover, we adopt the supremum of this relative error as the distortion measure $D_{f}$, which is given by

(4) $$\begin{array}{c} D_{f}\left(Sa_{i},S{\hat{a}}_{i}\right)=D_{f}\left(L_{i},l_{i}\right)=\frac{r^{L_{i}-l_{i}}-1}{r^{L_{i}}-1}. \end{array}$$

In particular, we obtain $D_{f}\left(L_{i},L_{i}\right)=0$ and $D_{f}\left(L_{i},0\right)=1$. Moreover, it is easy to check that $0\leq D_{f}\left(L_{i},l_{i}\right)\leq 1$ and $D_{f}\left(L_{i},l_{i}\right)$ decreases with the increasing of $l_{i}$. In fact, $D_{f}$ can be regarded as the percentage of data value loss in this case. Thus, the weighted reconstruction error in Equation (1) represents the total cost in data storage based on the loss degree.

In this stored procedure, the compression rate is $\left(\sum_{i=1}^{n}p_{i}l_{i}\right)/\left(\sum_{i=1}^{n}p_{i}L_{i}\right)$, and the total saving storage size is $\sum_{i=1}^{n}p_{i}\left(L_{i}-l_{i}\right)K$. Actually, *K* denotes the number of data, and it is extremely big due to the sharply increasing data deluge in the era of big data. Therefore, although $\left(L_{i}-l_{i}\right)$ is not always large, the saving storage size is still exceedingly substantial since *K* is exceedingly large.

Furthermore, to simplify the comparisons under different conditions, the weighted reconstruction error is also normalized to the relative weighted reconstruction error (RWRE). In fact, the RWRE characterizes the relative total cost in the data compression, and it is given by

(5) $$\begin{array}{c} D_{r}\left(x,W\right)=D_{r}\left(W,L,l\right)=\frac{D(x,W)}{\max_{l_{i}}D\left(x,W\right)}=\frac{\sum_{i=1}^{n}p_{i}W_{i}D_{f}\left(L_{i},l_{i}\right)}{\sum_{i=1}^{n}p_{i}W_{i}}=\frac{\sum_{i=1}^{n}p_{i}W_{i}\frac{r^{L_{i}-l_{i}}-1}{r^{L_{i}}-1}}{\sum_{i=1}^{n}p_{i}W_{i}}, \end{array}$$

where $L=\{L_{1},\ldots ,L_{n}\}$ and $l=\{l_{1},\ldots ,l_{n}\}$.

### 2.3. Problem Formulation

#### 2.3.1. General Storage System

In fact, the actual storage size of each data after the compression can then be expressed as $\sum_{i=1}^{n}p_{i}l_{i}$. For each given maximum available storage space constraint $\sum_{i=1}^{n}p_{i}l_{i}\leq T$, where *T* denotes the maximum available average storage size, we shall optimize the storage resources allocation strategy of this system by minimizing the RWRE, which can be expressed as

(6) $$\begin{array}{cc} P_{1}:\;\;\min_{l_{i}} & D_{r}\left(x,W\right) \end{array}$$

(6a) $$\begin{array}{cc} s.t. & \sum_{i=1}^{n}p_{i}l_{i}\leq T \end{array}$$

(6b) $$\begin{array}{c} 0\leq l_{i}\leq L_{i}\;\;\mathrm{for}\;\;i=1,2,\ldots ,n. \end{array}$$

The storage systems, which can be characterized by Problem $P_{1}$, are referred to as the *general storage system*.

**Remark** **1.** *In fact, this paper focuses on allocating resources by category with taking message importance into account, while the conventional source coding searches the shortest average description length of a random variable.*

#### 2.3.2. Ideal Storage System

In practice, the storage size of raw data is usually assigned to be the same for ease of use. Thus, we mainly consider the case where the original storage size of each data is the same, and use *L* to denote it (i.e., $L_{i}=L$ for i=1,2,…,n). As a result, we have

(7) $$\begin{array}{c} \min_{l_{i}}D_{r}\left(x,W\right)=\frac{r^{L}\min_{l_{i}}\sum_{i=1}^{n}p_{i}W_{i}r^{-l_{i}}}{\left(r^{L}-1\right)\sum_{i=1}^{n}p_{i}W_{i}}-\frac{1}{r^{L}-1}. \end{array}$$

Thus, the problem $P_{1}$ can be rewritten as

(8) $$\begin{array}{cc} P_{2}:\;\;\min_{l_{i}}\;\;\; & \sum_{i=1}^{n}p_{i}W_{i}r^{-l_{i}} \end{array}$$

(8a) $$\begin{array}{cc} s.t.\;\;\; & \sum_{i=1}^{n}p_{i}l_{i}\leq T \end{array}$$

(8b) $$\begin{array}{c} 0\leq l_{i}\leq L\;\;\mathrm{for}\;\;i=1,2,\ldots ,n. \end{array}$$

For convenience, we use the *ideal storage system* to represent the storage systems, which can be described by Problem $P_{2}$. Moreover, we will mainly focus on the characteristics of the solutions in Problem $P_{2}$ in this paper.

#### 2.3.3. Quantification Storage System

A *quantification storage system* quantizes and stores the real data acquired from sensors in the real world. The data is usually a real number, which requires an infinite number of bits to describe it accurately. That is, the original storage size of each class approaches the infinite number, (i.e., $L_{i}=L\to +\infty$ for i=1,2,…,n), in this case. As a result, the RWRE can be rewritten as

(9) $$\begin{array}{c} D_{r}\left(x,W\right)=\lim_{L\to \infty }\left\{\frac{\sum_{i=1}^{n}p_{i}W_{i}r^{-l_{i}}}{\left(1-r^{-L}\right)\sum_{i=1}^{n}p_{i}W_{i}}-\frac{1}{r^{L}-1}\right\}=\frac{\sum_{i=1}^{n}p_{i}W_{i}r^{-l_{i}}}{\sum_{i=1}^{n}p_{i}W_{i}}. \end{array}$$

Therefore, the problem $P_{1}$ in this case is reduced to

(10) $$\begin{array}{cc} P_{3}:\;\;\min_{l_{i}}\;\;\; & \sum_{i=1}^{n}p_{i}W_{i}r^{-l_{i}} \end{array}$$

(10a) $$\begin{array}{cc} s.t.\;\;\; & \sum_{i=1}^{n}p_{i}l_{i}\leq T \end{array}$$

(10b) $$\begin{array}{c} l_{i}\geq 0\;\;\mathrm{for}\;\;i=1,2,\ldots ,n. \end{array}$$

## 3. Optimal Allocation Strategy with Limited Storage Space

In this section, we shall first solve the problem $P_{1}$ and give the solutions. In fact, the solutions provide the optimal storage space allocation strategy for digital data on the best effort in minimizing the relative weighted reconstruction error (RWRE) when the total available storage size is limited. Then, the problem $P_{2}$ will be solved, the solutions of which characterize the optimal storage space allocation strategy with the same original storage size. Moreover, we shall also discuss the solutions in the case where the original storage size of each class approaches the infinite number by studying the problem $P_{3}$.

### 3.1. Optimal Allocation Strategy in General Storage System

**Theorem** **1.** *For a storage system with probability distribution $\left(p_{1},p_{2},\ldots ,p_{n}\right)$, $L_{i}$ is the storage size of the raw data of the class i for i=1,2,…,n. For a given maximum available average storage size T ($0\leq T\leq \sum_{i=1}^{n}p_{i}L_{i}$), when the radix is r (r>1), the solution of Problem $P_{1}$ is given by*

(11) $$l_{i}=\left\{\begin{array}{cc} 0 & \mathrm{if}l_{i}<0,\\ \frac{\ln\left(\ln r\right)+\ln W_{i}-\ln\left(1-r^{-L_{i}}\right)-\ln\lambda^{*}}{\ln r} & \mathrm{if}0\leq l_{i}\leq L_{i},\\ L_{i} & \mathrm{if}l_{i}>L_{i}, \end{array}\right.$$

where $\lambda^{*}$ is chosen so that $\sum_{i=1}^{n}p_{i}l_{i}=T$.

**Proof.**  By means of Lagrange multipliers and Karush–Kuhn–Tucher conditions, when ignoring the constant $\sum_{i=1}^{n}p_{i}W_{i}$, we set up the functional

(12) $$J=\sum_{i=1}^{n}p_{i}W_{i}\frac{r^{L_{i}-l_{i}}-1}{r^{L_{i}}-1}+\lambda^{*}\left(\sum_{i=1}^{n}p_{i}l_{i}-T\right)+\mu_{1}\left(l_{1}-L_{1}\right)+\ldots +\mu_{n}\left(l_{n}-L_{n}\right).$$

Differentiating with respect to $l_{i}$ and setting the derivative to zero, we have

(13) $$\begin{array}{cc} \frac{\partial J}{\partial l_{i}}=-p_{i}W_{i}\frac{r^{-l_{i}}}{1-r^{-L_{i}}}+\lambda^{*}p_{i}+\mu_{i}=0 & \;\;\mathrm{for}\;\;i=1,2,\ldots ,n\;\; \end{array}$$

(13a) $$\begin{array}{c} \sum_{i=1}^{n}p_{i}l_{i}-T=0 \end{array}$$

(13b) $$\begin{array}{cc} \mu_{i}\left(l_{i}-L_{i}\right)=0 & \;\;\mathrm{for}\;\;i=1,2,\ldots ,n\;\; \end{array}$$

(13c) $$\begin{array}{cc} l_{i}-L_{i}\leq 0 & \;\;\mathrm{for}\;\;i=1,2,\ldots ,n\;\; \end{array}$$

(13d) $$\begin{array}{cc} \mu_{i}\geq 0 & \;\;\mathrm{for}\;\;i=1,2,\ldots ,n\;\; \end{array}$$

(13e) $$\begin{array}{cc} l_{i}\geq 0 & \;\;\mathrm{for}\;\;i=1,2,\ldots ,n\;\; \end{array}$$

Hence, we obtain

(14) $$\begin{array}{c} l_{i}=\frac{\ln p_{i}+\ln\left(\ln r\right)+\ln W_{i}-\ln\left(1-r^{-L_{i}}\right)-\ln\left(\lambda^{*}p_{i}+\mu_{i}\right)}{\ln r}. \end{array}$$

First, it is easy to check that Equations (13b)–(13d) hold when $\mu_{i}=0$ and $l_{i}\leq L_{i}$. Hence, we have

(15) $$\begin{array}{c} l_{i}=\frac{\ln\left(\ln r\right)+\ln W_{i}-\ln\left(1-r^{-L_{i}}\right)-\ln\lambda^{*}}{\ln r}. \end{array}$$

Second, if $l_{i}$ in Equation (14) is larger than $L_{i}$, we will have $\mu_{i}>0$ and $l_{i}=L_{i}$ due to Equations (13b)–(13d).

Third, if $l_{i}<0$, we will let $l_{i}=0$ according to Equation (13e).

Moreover, $\lambda^{*}$ is chosen so that $\sum_{i=1}^{n}p_{i}l_{i}=T$ due to Equation (13a).

Therefore, based on the discussion above, we get Equation (11) in order to ensure $0\leq l_{i}\leq L_{i}$. □

**Remark** **2.** Let $\tilde{N}$ be the number of $l_{i}$ which meets $0\leq l_{i}\leq L_{i}$ and $\left\{I_{j},j=1,2,\ldots ,\tilde{N}\right\}$ is part of the sequence of {1,2,…,N} which satisfies $0\leq \ln\left(\ln r\right)+\ln W_{I_{j}}-\ln\left(1-r^{-L_{I_{j}}}\right)-\ln\lambda^{*}\leq L_{I_{j}}\ln r$. Furthermore, $\left\{T_{j},j=1,2,\ldots ,{\tilde{N}}_{L}\right\}$ is used to denote the part of the sequence of {1,2,…,N} which satisfies $\ln\left(\ln r\right)+\ln W_{T_{j}}-\ln\left(1-r^{-L_{T_{j}}}\right)-\ln\lambda^{*}>L_{T_{j}}\ln r$.

Substituting Equation (11) in the constraint $\sum_{i=1}^{n}p_{i}l_{i}=T$, we have

(16) $$\begin{array}{c} \ln\lambda^{*}=\ln\ln r+\frac{\sum_{j=1}^{\tilde{N}}p_{I_{j}}\ln W_{I_{j}}-\sum_{j=1}^{\tilde{N}}p_{I_{j}}\ln\left(1-r^{-L_{I_{j}}}\right)-\ln r\left(T-\sum_{j=1}^{{\tilde{N}}_{L}}p_{T_{j}}L_{T_{j}}\right)}{\sum_{j=1}^{\tilde{N}}p_{I_{j}}}. \end{array}$$

Hence, for $0\leq l_{i}\leq L$, we obtain

(17) $$\begin{array}{c} l_{i}=\frac{T-\sum_{j=1}^{{\tilde{N}}_{L}}p_{T_{j}}L_{T_{j}}}{\sum_{j=1}^{\tilde{N}}p_{I_{j}}}+\frac{\ln W_{i}}{\ln r}-\frac{\sum_{j=1}^{\tilde{N}}p_{I_{j}}\ln W_{I_{j}}}{\ln r\sum_{j=1}^{\tilde{N}}p_{I_{j}}}+\frac{\sum_{j=1}^{\tilde{N}}p_{I_{j}}\ln\left(1-r^{-L_{I_{j}}}\right)}{\ln r\sum_{j=1}^{\tilde{N}}p_{I_{j}}}. \end{array}$$

In fact, *T*, $p_{i}$, *r*, $L_{i}$ are usually constraints for a given storage system, and therefore $l_{i}$ is only determined by the second and the third items on the right side of Equation (17), which means the storage size depends on the message importance and the probability distribution of class for the given available storage size.

**Remark** **3.** Since the actual compressed storage size $l_{i}^{*}$ must be an integer, the actual storage size allocation strategy is

(18) $$\begin{array}{c} l_{i}^{*}=\min\left({\left\lfloor \frac{T-\sum_{j=1}^{{\tilde{N}}_{L}}p_{T_{j}}L_{T_{j}}}{\sum_{j=1}^{\tilde{N}}p_{I_{j}}}+\frac{\ln W_{i}}{\ln r}-\frac{\sum_{j=1}^{\tilde{N}}p_{I_{j}}\ln W_{I_{j}}}{\ln r\sum_{j=1}^{\tilde{N}}p_{I_{j}}}+\frac{\sum_{j=1}^{\tilde{N}}p_{I_{j}}\ln\left(1-r^{-L_{I_{j}}}\right)}{\ln r\sum_{j=1}^{\tilde{N}}p_{I_{j}}}\right\rfloor }^{+},L_{i}\right), \end{array}$$

*where ${\left(x\right)}^{+}$ is equal to x when x≥0, and it is zero when x<0. In addition, ⌊x⌋ is the largest integer smaller than or equal to x.*

### 3.2. Optimal Allocation Strategy in Ideal Storage System

Then, we pay attention to the case where the original storage size of each data is the same. Based on Theorem 1, we get the following corollary in the ideal storage system.

**Corollary** **1.** *For a storage system with probability distribution $\left(p_{1},p_{2},\ldots ,p_{n}\right)$, the original storage size of each class is the same, which is given by $L_{i}=L$ for i=1,2,…,n. For a given maximum available average storage size T (0≤T≤L), when the radix is r (r>1), the solution of Problem $P_{2}$ is given by*

(19) $$l_{i}=\left\{\begin{array}{cc} 0 & if\;l_{i}<0,\\ \frac{\ln\left(\ln r\right)+\ln W_{i}-\ln\lambda }{\ln r} & if\;0\leq l_{i}\leq L,\\ L & if\;l_{i}>L, \end{array}\right.$$

*where λ is chosen so that $\sum_{i=1}^{n}p_{i}l_{i}=T$.*

**Proof.**  Let $\lambda =\lambda^{*}\left(1-r^{-L}\right)$ and $L_{i}=L$ for i=1,2,…,n. Substituting them in Equation (11), we find that $l_{i}$ in this case can be rewritten as Equation (19). □

Substituting Equation (19) in the constraint $\sum_{i=1}^{n}p_{i}l_{i}=T$, we obtain

(20) $$\begin{array}{c} \ln\lambda =\ln\ln r+\frac{\sum_{j=1}^{\tilde{N}}p_{I_{j}}\ln W_{I_{j}}-\ln r\left(T-T_{N_{L}}\right)}{\sum_{j=1}^{\tilde{N}}p_{I_{j}}}, \end{array}$$

where $\tilde{N}$, ${\tilde{N}}_{L}$, $I_{j}$, $T_{j}$ is still given by Remark 2 with letting $\lambda =\lambda^{*}\left(1-r^{-L}\right)$. In addition, $T_{N_{L}}=\sum_{j=1}^{{\tilde{N}}_{L}}p_{T_{j}}L$. Hence, for $0\leq l_{i}\leq L$, we obtain

(21) $$\begin{array}{c} l_{i}=\frac{T-T_{N_{L}}}{\sum_{j=1}^{\tilde{N}}p_{I_{j}}}+\frac{\ln W_{i}}{\ln r}-\frac{\sum_{j=1}^{\tilde{N}}p_{I_{j}}\ln W_{I_{j}}}{\ln r\sum_{j=1}^{\tilde{N}}p_{I_{j}}}. \end{array}$$

**Remark** **4.** Since the actual compressed storage size $l_{i}^{*}$ must be an integer, the actual storage size allocation strategy is

(22) $$\begin{array}{c} l_{i}^{*}=\min\left({\left\lfloor \frac{T-T_{N_{L}}}{\sum_{j=1}^{\tilde{N}}p_{I_{j}}}+\frac{\ln W_{i}}{\ln r}-\frac{\sum_{j=1}^{\tilde{N}}p_{I_{j}}\ln W_{I_{j}}}{\ln r\sum_{j=1}^{\tilde{N}}p_{I_{j}}}\right\rfloor }^{+},L\right). \end{array}$$

**Remark** **5.** *When $\tilde{N}=n$, $0\leq l_{i}\leq L$ always holds for 1≤i≤n, and the actual storage size is given by*

(23) $$\begin{array}{c} l_{i}^{*}=\left\lfloor T+\frac{\ln W_{i}-\sum_{i=1}^{n}p_{i}\ln W_{i}}{\ln r}\right\rfloor . \end{array}$$

In order to illustrate the geometric interpretation of this algorithm, let

(24) $$\begin{array}{c} \beta =\frac{\ln\ln r-\ln\lambda }{\ln r}. \end{array}$$

Hence, the optimal storage size can be simplified to

(25) $$\begin{array}{cc} l_{i}= & \left\{\begin{array}{c} \;\;0,\;\;\;\;\;\;\;\;\;\;\;\mathrm{if}\;\;\beta -\frac{\ln(1/W_{i})}{\ln r}<0.\\ \beta -\frac{\ln(1/W_{i})}{\ln r}\;\;\;\;\;\;\mathrm{if}\;\;0\leq \beta -\frac{\ln(1/W_{i})}{\ln r}\leq L.\\ \;\;L,\;\;\;\;\;\;\;\;\;\;\;\mathrm{if}\;\;\beta -\frac{\ln(1/W_{i})}{\ln r}>L. \end{array}\right. \end{array}$$

The monotonicity of optimal storage size with respect to importance weight is discussed in the following theorem.

**Theorem** **2.** *Let $\left(p_{1},p_{2},\ldots ,p_{n}\right)$ be a probability distribution and $W=W_{1},\ldots ,W_{n}$ be importance weights. L and r are fixed positive integers (r>1). The solution of Problem $P_{2}$ meets: $l_{i}\geq l_{j}$ if $W_{i}>W_{j}$ for ∀i,j∈{1,2,…,n}.*

**Proof.**  Refer to the Appendix A. □

**Figure 2:**
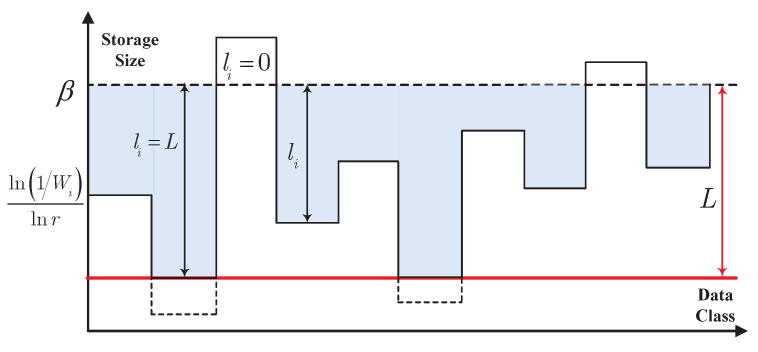
Restrictive water-filling for optimal storage sizes.

This gives rise to a kind of restrictive water-filling, which is presented in Figure 2. Choose a constant β so that $\sum_{i=1}^{n}p_{i}l_{i}=T$. The storage size depends on the difference between β and $\frac{\ln(1/W_{i})}{\ln r}$. In Figure 2, we obtain that β characterizes the height of water surface, and $\frac{\ln(1/W_{i})}{\ln r}$ determines the bottom of the pool. Actually, no storage space is assigned to the data when this difference is less than zero. When the difference is in the interval [0,L], this difference is exactly the storage size. Furthermore, the storage size will be truncated to *L* bits if the difference is larger than *L*. Compared with the conventional water-filling, the lowest height of the bottom of the pool is constricted in this restrictive water-filling.

**Remark** **6.** *The restrictive water-filling in Figure 2 is summarized as follows.*

- *For the data with extremely small message importance, $\frac{\ln(1/W_{i})}{\ln r}$ is so large that the bottom of the pool is above the water surface. Thus, the storage size of this kind of data is zero.*
- *For the data with small message importance, $\frac{\ln(1/W_{i})}{\ln r}$ is large, and therefore the bottom of the pool is high. Thus, the storage size of this kind of data is small.*
- *For the data with large message importance, $\frac{\ln(1/W_{i})}{\ln r}$ is small, and therefore the bottom of the pool is low. Thus, the storage size of this kind of data is large.*
- *For the data with extremely large message importance, $\frac{\ln(1/W_{i})}{\ln r}$ is so small that the bottom of the pool is constricted in order to truncate the storage size to L.*

Thus, this optimal storage space allocation strategy is a high efficient adaptive storage allocation algorithm for the fact that it can make rational use of all the storage space according to message importance to minimize the RWRE.

This solution can be gotten by means of the recursive algorithm in practice, which is shown in Algorithm 1, where we define an auxiliary function as

(26) $$\begin{array}{cc} f(i,W,P,L,T,r,K_{\min},K_{\max}) & =\left\{\begin{array}{cc} L & \mathrm{if}\;\;1\leq i<K_{\min}.\\ \frac{T-\sum_{j=1}^{K_{\min}-1}p_{j}L}{\sum_{j=K_{\min}}^{K_{\max}}p_{j}}+\frac{\ln W_{i}}{\ln r}-\frac{\sum_{j=K_{\min}}^{K_{\max}}p_{j}\ln W_{j}}{\ln r\sum_{j=K_{\min}}^{K_{\max}}p_{j}} & \mathrm{if}\;\;K_{\min}\leq i\leq K_{\max}.\\ 0 & \mathrm{if}\;\;K_{\max}<i\leq n. \end{array}\right. \end{array}$$

### 3.3. Optimal Allocation Strategy in Quantification Storage System

**Corollary** **2.** *For a given maximum available average storage size T (T≥0), when probability distribution is $\left(p_{1},p_{2},\ldots ,p_{n}\right)$ and the radix is r (r>1), the solution of Problem $P_{3}$ is given by*

(27) $$\begin{array}{c} l_{i}={\left(\frac{\ln\left(\ln r\right)+\ln W_{i}-\ln\lambda }{\ln r}\right)}^{+}, \end{array}$$

*where λ is chosen so that $\sum_{i=1}^{n}p_{i}l_{i}=T$.*

**Proof.**  Let L→∞ in Corollary 1, the solutions in Equation (19) can be simplified to Equation (27). □

In fact, the optimal storage space allocation strategy in this case can be seen as a kind of water-filling, which gets rid of the constraint on the lowest height of the bottom of the pool.
**Algorithm 1** Storage Space Allocation Algorithm**Require:**  The message importance, $W=\{W_{i},i=1,2,\ldots ,n\}$ (Sort it to satisfy $W_{1}\geq W_{2}\geq \ldots \geq W_{n}$)  The probability distribution of source, $P=\{p_{i},i=1,2,\ldots ,n\}$  The original storage size, *L* and $L=\left\{L_{i}=L,i=1,2,\ldots ,n\right\}=\left\{L,\ldots ,L\right\}$  The maximum available average storage size, *T*  The radix, *r*  The auxiliary variables, $K_{\min},K_{\max}$ (Let $K_{\min}=1,K_{\max}=n$ as the original values)**Ensure:**  The compressed storage size, $l=\{l_{i},i=1,\ldots ,n\}$  Denote this recursive algorithm as $\phi (W,P,L,T,r,K_{\min},K_{\max})$1:${l_{i}}^{\prime }\leftarrow f\left(i,W,P,L,T,r,K_{\min},K_{\max}\right)$ for i=1,…,n                           ⊳ See Equation (26)2:**if**∀t∈{1,…,n} such that $0\leq l_{t}^{\prime }\leq L$ and $\sum_{i=1}^{n}p_{i}l_{i}^{\prime }=T$3:$l_{i}\leftarrow {l_{i}}^{\prime }$ for i=1,…,n4:**else**    **if** $K_{\max}>K_{\min}$5:         $l^{\left(1\right)}\leftarrow \phi \left(W,P,L,T,r,K_{\min},K_{\max}-1\right)$   (Make a recursive call with $K_{\max}\leftarrow K_{\max}-1$)6:         $\epsilon^{\left(1\right)}=D_{r}\left(W,L,l^{\left(1\right)}\right)$          (Calculate the RWRE with $l^{\left(1\right)}$)     ⊳ See Equation (5)7:         $l^{\left(2\right)}\leftarrow \phi \left(W,P,L,T^{\prime },r,K_{\min}+1,K_{\max}\right)$   (Make a recursive call with $K_{\min}\leftarrow K_{\min}+1$)8:         $\epsilon^{\left(2\right)}=D_{r}\left(W,L,l^{\left(2\right)}\right)$          (Calculate the RWRE with $l^{\left(2\right)}$)     ⊳ See Equation (5) 9:               **if** $\epsilon^{\left(1\right)}\leq \epsilon^{\left(2\right)}$10:                     $l\leftarrow l^{\left(1\right)}$11:               **else**12:                     $l\leftarrow l^{\left(2\right)}$13:               **end**14:         **else**15:         $l_{K_{\min}}\leftarrow \left(T-\sum_{i=1}^{K_{\min}-1}p_{i}L\right)/p_{K_{\min}}$, $l_{i}\leftarrow L$ when $i<K_{\min}$, $l_{i}\leftarrow 0$ when $i>K_{\min}$16:         **end**17:**end**18:**return**l

## 4. Property of Optimal Storage Strategy Based on Message Importance Measure

Considering that the ideal storage system can capture most of the characteristics of the lossy compression storage model in this paper, we focus on the properties of optimal storage strategy in it in this section for ease of analysis. Specifically, we ignore rounding and adopt $l_{i}$ in Equation (19) as the optimal storage size of the *i*-th class in this section. Moreover, we focus on a special kind of the importance weight. Namely, the message importance measure (MIM) is adopted as the importance weight in this part, for the fact that it can effectively measure the cost of the error in data reconstruction in the small-probability event scenarios [23,31].

### 4.1. Normalized Message Importance Measure

In order to facilitate comparison under different parameters, the normalized MIM is used and we can write

(28) $$\begin{array}{c} W_{i}=\frac{e^{\varpi (1-p_{i})}}{\sum_{j=1}^{n}e^{\varpi (1-p_{j})}}, \end{array}$$

where ϖ is the importance coefficient, whose selection is discussed in Reference [37]. In fact, the MIM characterizes the user’s subjective concern degree of data, and ϖ is an indicator that reflects the user preferences. In practice, the values of ϖ depend on the user preferences. For instance, when ϖ is positive, the user only focuses on the small-probability events, while the large-probability events are focused on when ϖ is negative [33].

Actually, it is easy to check that $0\leq W_{i}\leq 1$ for i=1,2,…,n. Moreover, it is obvious that the sum of those in all event classes is one.

#### 4.1.1. Positive Importance Coefficient

For positive importance coefficient (i.e., ϖ>0), let $\alpha_{1}=\arg\min_{i}p_{i}$ and assume $p_{\alpha_{1}}<p_{i}$ for $i\neq \alpha_{1}$. The derivative of it with respect to the importance coefficient is

(29) $$\begin{array}{c} \frac{\partial W_{\alpha_{1}}}{\partial \varpi }=\frac{\sum_{j=1}^{n}\left(p_{j}-p_{\alpha_{1}}\right)e^{\varpi (2-p_{\alpha_{1}}-p_{j})}}{{\left(\sum_{j=1}^{n}e^{\varpi (1-p_{j})}\right)}^{2}}\geq 0. \end{array}$$

Therefore, $W_{\alpha_{1}}$ increases as ϖ increases. In particular, as ϖ approaches positive infinity, we have

(30) $$\begin{array}{cc} \lim_{\varpi \to +\infty }W_{\alpha_{1}}= & \lim_{\varpi \to +\infty }\frac{e^{\varpi (1-p_{\alpha_{1}})}}{\sum_{j=1}^{n}e^{\varpi (1-p_{j})}} \end{array}$$

(30a) $$\begin{array}{cc} = & \lim_{\varpi \to +\infty }\frac{e^{\varpi (1-p_{\alpha_{1}})}}{e^{\varpi (1-p_{\alpha_{1}})}+\sum_{j\neq \alpha_{1}}e^{\varpi (1-p_{j})}} \end{array}$$

(30b) $$\begin{array}{cc} = & \lim_{\varpi \to +\infty }\frac{1}{1+\sum_{j\neq \alpha_{1}}e^{\varpi (p_{\alpha_{1}}-p_{j})}} \end{array}$$

(30c) $$\begin{array}{cc} = & 1. \end{array}$$

Obviously, $\lim_{\varpi \to +\infty }W_{i}=0$ for $i\neq \alpha_{1}$.

**Remark** **7.** *As ϖ approaches positive infinity, the importance weight with the smallest probability is one and others are all zero, which means only a fraction of data almost contains all of the critical information that users care about in the viewpoint of this message importance.*

#### 4.1.2. Negative Importance Coefficient

When the importance coefficient is negative (i.e., ϖ<0), let $\alpha_{2}=\arg\max_{i}p_{i}$ and assume $p_{\alpha_{2}}>p_{i}$ for $i\neq \alpha_{2}$. Its derivative with respect to the importance coefficient is

(31) $$\begin{array}{c} \frac{\partial W_{\alpha_{2}}}{\partial \varpi }=\frac{\sum_{j=1}^{n}\left(p_{j}-p_{\alpha_{2}}\right)e^{\varpi (2-p_{\alpha_{2}}-p_{j})}}{{\left(\sum_{j=1}^{n}e^{\varpi (1-p_{j})}\right)}^{2}}\leq 0. \end{array}$$

Therefore, $W_{\alpha_{2}}$ decreases as ϖ increases. In particular, as ϖ approaches negative infinity, we have

(32) $$\begin{array}{cc} \lim_{\varpi \to -\infty }W_{\alpha_{2}}= & \lim_{\varpi \to -\infty }\frac{e^{\varpi (1-p_{\alpha_{2}})}}{\sum_{j=1}^{n}e^{\varpi (1-p_{j})}} \end{array}$$

(32a) $$\begin{array}{cc} = & \lim_{\varpi \to -\infty }\frac{e^{\varpi (1-p_{\alpha_{2}})}}{e^{\varpi (1-p_{\alpha_{2}})}+\sum_{j\neq \alpha_{2}}e^{\varpi (1-p_{j})}} \end{array}$$

(32b) $$\begin{array}{cc} = & \lim_{\varpi \to -\infty }\frac{1}{1+\sum_{j\neq \alpha_{2}}e^{\varpi (p_{\alpha_{2}}-p_{j})}} \end{array}$$

(32c) $$\begin{array}{cc} = & 1. \end{array}$$

Obviously, $\lim_{\varpi \to -\infty }W_{i}=0$ for $i\neq \alpha_{2}$.

**Remark** **8.** *As ϖ approaches negative infinity, the importance weight with the biggest probability is one and others are all zero. If the biggest probability is far from 1, the majority of message importance can also be included in those data with the highest probability, and the corresponding part of the data is not too much.*

### 4.2. Optimal Storage Size for Each Class

Assume $\tilde{N}=n$ and ignore rounding, due to Equation (23), we obtain

(33) $$\begin{array}{cc} l_{i}= & T+\frac{\ln\frac{e^{\varpi (1-p_{i})}}{\sum_{j=1}^{n}e^{\varpi (1-p_{j})}}-\sum_{i=1}^{n}p_{i}\ln\frac{e^{\varpi (1-p_{i})}}{\sum_{j=1}^{n}e^{\varpi (1-p_{j})}}}{\ln r}\\ = & T+\frac{\varpi }{\ln r}\left(\gamma_{p}-p_{i}\right), \end{array}$$

where $\gamma_{p}$ is an auxiliary variable and it is given by

(34) $$\begin{array}{c} \gamma_{p}=\sum_{i=1}^{n}p_{i}^{2}. \end{array}$$

In fact, it is a functional of the minus Rényi entropy of order two, i.e., $\gamma_{p}=e^{-H_{2}\left(P\right)}$ where $H_{2}\left(P\right)$ is the Rényi entropy $H_{\alpha }\left(\cdot \right)$ when α=2 [38]. Furthermore, we have the following lemma on $\gamma_{p}$.

**Lemma** **1.** *Let $\left(p_{1},p_{2},\ldots ,p_{n}\right)$ be a probability distribution, then we have*

(35) $$\begin{array}{cc} \frac{1}{n} & \leq \gamma_{p}\leq 1, \end{array}$$

(35a) $$\begin{array}{cc} -\frac{1}{4} & \leq \gamma_{p}-p_{i}\leq 1. \end{array}$$

**Proof.**  Refer to Appendix B. □

Thus, we find $l_{i}>T$ if $(1/n-p_{i})\varpi >0$. Furthermore, we obtain $l_{i}=T$ when $p_{i}=\gamma_{p}$.

**Theorem** **3.** *Let $\left(p_{1},p_{2},\ldots ,p_{n}\right)$ be a probability distribution and $W_{i}=e^{\varpi (1-p_{i})}/\sum_{j=1}^{n}e^{\varpi (1-p_{j})}$ be the importance weight. The optimal storage sizes in the ideal storage system have the following properties:*

*(1)*  *$l_{i}\geq l_{j}$ if $p_{i}<p_{j}$ for ∀i,j∈{1,2,…,n} when ϖ>0;*
*(2)*  *$l_{i}\leq l_{j}$ if $p_{i}<p_{j}$ for ∀i,j∈{1,2,…,n} when ϖ<0.*

**Proof.**  Refer to Appendix C. □

**Remark** **9.** *As noted in [31], the data with smaller probability usually possesses larger importance when ϖ>0, while the data with larger probability usually possesses larger importance when ϖ<0. Therefore, this optimal allocation strategy makes rational use of all the storage space by providing more storage size for the paramount data and less storage size for the insignificance data. It agrees with the intuitive idea, which is that users generally are more concerned about the data that they need rather than the whole data itself.*

**Lemma** **2.** *Let $\left(p_{1},p_{2},\ldots ,p_{n}\right)$ be a probability distribution and r be radix. L and T are positive integers, and T<L. If ϖ meets $0\leq T+\varpi (\gamma_{p}-p_{i})/\ln r\leq L$, then we have $\tilde{N}=n$.*

**Proof.**  According to Equation (33a) and constraint $0\leq T+\varpi (\gamma_{p}-p_{i})/\ln r\leq L$, we obtain $0\leq l_{i}\leq L$ for ∀i∈{1,2,…,n}. In this case, $\tilde{N}=n$. □

In fact, when ϖ≥0, due to Equation (33a) and Lemma 1, we obtain

(36) $$\begin{array}{cc} 0\leq T-\frac{\varpi }{4\ln r}\leq T+\frac{\varpi (\gamma_{p}-p_{i})}{\ln r} & \leq T+\frac{\varpi }{\ln r}\leq L. \end{array}$$

Similarly, when ϖ<0, we have

(37) $$\begin{array}{cc} 0\leq T+\frac{\varpi }{\ln r}\leq T+\frac{\varpi (\gamma_{p}-p_{i})}{\ln r} & \leq T-\frac{\varpi }{4\ln r}\leq L. \end{array}$$

According to Equations (36) and (37), we find $\tilde{N}=n$ always holds if max(4lnr(T−L),−T/lnr)≤ϖ≤min(4Tlnr,lnr(L−T)).

### 4.3. Relative Weighted Reconstruction Error

For convenience, we also use D(x,ϖ) to denote the relative weighted reconstruction error (RWRE) D(x,W). Due to Equation (7), we have

(38) $$\begin{array}{c} D_{r}\left(x,\varpi \right)=\frac{1}{r^{L}-1}\left(\frac{\sum_{i=1}^{n}p_{i}e^{\varpi (1-p_{i})}r^{L-l_{i}}}{\sum_{i=1}^{n}p_{i}e^{\varpi (1-p_{i})}}-1\right). \end{array}$$

If the maximum available average storage size *T* is zero, then we will have $l_{i}=0$ for i=1,2,…,n. In this case, $D_{r}\left(x,\varpi \right)=1$. On the contrary, $D_{r}\left(x,\varpi \right)=0$ when $l_{i}=L$ for i=1,2,…,n.

**Theorem** **4.** *$D_{r}\left(x,\varpi \right)$ has the following properties:*

*(1)*  *$D_{r}\left(x,\varpi \right)$ is monotonically decreasing with ϖ in (0,+∞);*
*(2)*  *$D_{r}\left(x,\varpi \right)$ is monotonically increasing with ϖ in (−∞,0);*
*(3)*  *$D_{r}\left(x,\varpi \right)\leq D_{r}\left(x,0\right)=\left(r^{L-T}-1\right)/\left(r^{L}-1\right)$.*

**Proof.**  Refer to Appendix D. □

**Remark** **10.** *As shown in Remark 7 and Remark 8, the overwhelming majority of important information will gather in a fraction of data as the importance coefficient increases to negative/positive infinity. Therefore, we can heavily reduce the storage space with extremely small of RWRE with the increasing of the absolute value of the importance coefficient. In fact, this special characteristic of weight reflects the effect of users’ preferences. That is, it is beneficial for data compression that the data that users care about is highly clustered. Moreover, when ϖ=0, all the importance weights are the same, which leads to the incompressibility, in a sense, for the fact that there is no special characteristic of weight for users to make rational use of storage space.*

In the following part of this section, we will discuss the cases where $0\leq T+\varpi (\gamma_{p}-p_{i})/\ln r\leq L$ for i=1,…,n, which means all $l_{i}$ can be given by Equation (33a) and $n=\tilde{N}$ due to Lemma 2. In this case, substituting Equation (33a) in Equation (7), the RWRE is

(39) $$\begin{array}{c} D_{r}\left(x,\varpi \right)=\frac{e^{\varpi (1-\gamma_{p})}r^{\Delta }}{\left(r^{L}-1\right)\sum_{i=1}^{n}p_{i}e^{\varpi (1-p_{i})}}-\frac{1}{r^{L}-1}, \end{array}$$

where Δ=L−T, which characterizes the average compressed storage space of each data.

Since $0\leq T+\varpi (\gamma_{p}-p_{i})/\ln r\leq L$, we have

(40) $$\begin{array}{c} \left\{\begin{array}{cc} \frac{\varpi (\gamma_{p}-p_{\alpha_{1}})}{\ln r}\leq L-T\leq L+\frac{\varpi (\gamma_{p}-p_{\alpha_{2}})}{\ln r} & \;\mathrm{if}\;\;\;\varpi \geq 0.\\ \frac{\varpi (\gamma_{p}-p_{\alpha_{2}})}{\ln r}\leq L-T\leq L+\frac{\varpi (\gamma_{p}-p_{\alpha_{1}})}{\ln r} & \;\mathrm{if}\;\;\;\varpi <0. \end{array}\right. \end{array}$$

Hence,

(41) $$\begin{array}{c} \delta_{1}\leq D_{r}\left(x,\varpi \right)\leq \delta_{2}. \end{array}$$

where

(42) $$\begin{array}{c} \delta_{1}=\left\{\begin{array}{cc} \frac{e^{\varpi (1-p_{\alpha_{1}})}}{\left(r^{L}-1\right)\sum_{i=1}^{n}p_{i}e^{\varpi (1-p_{i})}}-\frac{1}{r^{L}-1} & \;\mathrm{if}\;\;\;\varpi \geq 0,\\ \frac{e^{\varpi (1-p_{\alpha_{2}})}}{\left(r^{L}-1\right)\sum_{i=1}^{n}p_{i}e^{\varpi (1-p_{i})}}-\frac{1}{r^{L}-1} & \;\mathrm{if}\;\;\;\varpi <0, \end{array}\right. \end{array}$$

and

(43) $$\begin{array}{c} \delta_{2}=\left\{\begin{array}{cc} \frac{e^{\varpi (1-p_{\alpha_{2}})}r^{L}}{\left(r^{L}-1\right)\sum_{i=1}^{n}p_{i}e^{\varpi (1-p_{i})}}-\frac{1}{r^{L}-1} & \;\mathrm{if}\;\;\;\varpi \geq 0.\\ \frac{e^{\varpi (1-p_{\alpha_{1}})}r^{L}}{\left(r^{L}-1\right)\sum_{i=1}^{n}p_{i}e^{\varpi (1-p_{i})}}-\frac{1}{r^{L}-1} & \;\mathrm{if}\;\;\;\varpi <0. \end{array}\right. \end{array}$$

**Theorem** **5.** For a given storage system with the probability distribution of data sequence $P=(p_{1},p_{2},\ldots ,p_{n})$, let L, r be fixed positive integers (r>1), and ϖ meets $0\leq T+\varpi (\gamma_{p}-p_{i})/\ln r\leq L$ for i=1,2,…,n. For the given least upper bound of the RWRE δ ($\delta_{1}\leq \delta \leq \delta_{2}$ where $\delta_{1}$ and $\delta_{1}$ is defined in Equation (41)), the maximum average compressed storage size of each data $\Delta^{*}\left(\delta \right)$ is given by

(44) $$\begin{array}{cc} \Delta^{*}\left(\delta \right) & =\frac{\ln\left(1+\delta (r^{L}-1)\right)+L\left(\varpi ,P\right)-\varpi +\varpi \gamma_{p}}{\ln r} \end{array}$$

(44a) $$\begin{array}{c} \geq \frac{\ln\left(1+\delta (r^{L}-1)\right)}{\ln r}, \end{array}$$

*where $L\left(\varpi ,P\right)=\ln\sum_{i=1}^{n}p_{i}e^{\varpi (1-p_{i})}$, and the equality of Equation (44a) holds if the probability distribution of the data sequence is a uniform distribution or the importance coefficient is zero.*

**Proof.**  It is easy to check that $\tilde{N}=n$ according to Lemma 2 for the fact that $0\leq T+\varpi (\gamma_{p}-p_{i})/\ln r\leq L$. Let D(x,ϖ)≤δ. By means of Equation (39), we solve this inequality and obtain

(45) $$\begin{array}{c} \Delta \leq \frac{\ln\left(1+\delta (r^{L}-1)\right)+L\left(\varpi ,P\right)-\varpi +\varpi \gamma_{p}}{\ln r}=\Delta^{*}\left(\delta \right), \end{array}$$

where $L\left(\varpi ,P\right)=\ln\sum_{i=1}^{n}p_{i}e^{\varpi (1-p_{i})}$. Then we have the following inequality:

$$\begin{array}{c} \Delta^{*}\left(\delta \right)\overset{\left(a\right)}{\geq }\frac{\ln\left(1+\delta (r^{L}-1)\right)+\ln e^{\sum_{i=1}^{n}p_{i}\varpi \left(1-p_{i}\right)}-\varpi +\varpi \gamma_{p}}{\ln r}=\frac{\ln\left(1+\delta (r^{L}-1)\right)}{\ln r}, \end{array}$$

where (a) follows from Jensen’s inequality. Since the exponential function is strictly convex, the equality holds only if $\varpi (1-p_{i})$ is constant everywhere, which means $\left(p_{1},p_{2},\ldots ,p_{n}\right)$ is a uniform distribution or the importance coefficient ϖ is zero. □

**Remark** **11.** *In conventional source coding, the encoding length depends on the entropy of sequence, and a sequence is incompressible if its probability distribution is a uniform distribution [34]. In Theorem 5, the uniform distribution is also the worst case, since the system achieves the minimum compressed storage size. Although the focus is different, they both show that the uniform distribution is detrimental for compression.*

Furthermore, taking ϖ>0 as an example, it is also noted that

(46) $$\begin{array}{c} \Delta^{*}\left(\delta \right)\leq \Delta^{*}\left(\delta_{2}\right)=L+\frac{\varpi (\gamma_{p}-p_{\alpha_{2}})}{\ln r}\leq L, \end{array}$$

for the fact that $\gamma_{p}\leq p_{\alpha_{2}}$. In order to make $\Delta^{*}\left(\delta_{2}\right)$ approaches *L*, $\gamma_{p}-p_{\alpha_{2}}$ should be as close to zero as possible in the range where $0\leq T+\varpi (\gamma_{p}-p_{i})/\ln r\leq L$ for i=1,2,…,n holds.

When the importance coefficient is constant, for two probability distributions P and Q, if $L\left(\varpi ,P\right)+\varpi \gamma_{p}>L\left(\varpi ,Q\right)+\varpi \gamma_{q}$, then we will obtain $\Delta^{*}$ in P is larger than that in Q. In fact, L(ϖ,P) is defined as MIM in [30], and $\gamma_{p}=e^{-H_{2}\left(P\right)}$ [38]. Thus, the maximum average compressed storage size of each data is under the control of MIM and Rényi entropy of order two. For typical small-probability event scenarios where there is an exceedingly small probability, the MIM is usually large, and $\gamma_{p}$ is also not small simultaneously with big probability. Therefore, $\Delta^{*}\left(\delta \right)$ is usually large in this case. As a result, much more compressed storage space can be saved in typical small-probability event scenarios while compared to that in uniform probability distribution. Namely, the data can be compressed by means of the characteristic of the typical small-probability events, which may help to improve the design of practical storage systems in big data.

## 5. Property of Optimal Storage Strategy Based on Non-Parametric Message Importance Measure

In this section, we define the importance weight based on the form of non-parametric message importance measure (NMIM) to characterize the relative weighted reconstruction error (RWRE) [23]. Then, the importance weight of *i*-th class in this section is given by

(47) $$\begin{array}{c} W_{i}=\frac{e^{\left(1-p_{i}\right)/p_{i}}}{\sum_{j=1}^{n}e^{\left(1-p_{j}\right)/p_{j}}}. \end{array}$$

Due to Equation (22), the optimal storage size in the ideal storage system by this importance weight is given by

(48) $$\begin{array}{cc} l_{i}^{*} & =\min\left({\left\lfloor \frac{T-T_{N_{L}}}{\sum_{j=1}^{\tilde{N}}p_{I_{j}}}+\frac{1}{p_{i}\ln r}-\frac{1}{\ln r}-\frac{\ln\sum_{j=1}^{n}e^{\left(1-p_{j}\right)/p_{j}}}{\ln r}-\frac{\sum_{j=1}^{\tilde{N}}\left(1-p_{I_{j}}-p_{I_{j}}\ln\sum_{j=1}^{n}e^{\left(1-p_{j}\right)/p_{j}}\right)}{\ln r\sum_{j=1}^{\tilde{N}}p_{I_{j}}}\right\rfloor }^{+},L\right)\\ & =\min\left({\left\lfloor \frac{T-T_{N_{L}}}{\sum_{j=1}^{\tilde{N}}p_{I_{j}}}+\frac{1}{p_{i}\ln r}-\frac{\tilde{N}}{\ln r\sum_{j=1}^{\tilde{N}}p_{I_{j}}}\right\rfloor }^{+},L\right). \end{array}$$

For two probabilities $p_{i}$ and $p_{j}$, if $p_{i}<p_{j}$, then we will have $W_{i}>W_{j}$. In this case, we obtain $l_{i}^{*}\geq l_{j}^{*}$ according to Theorem 2.

Assume $\tilde{N}=n$ and ignore rounding, due to Equation (23), we obtain

(49) $$\begin{array}{c} l_{i}=T+\frac{1}{p_{i}\ln r}-\frac{n}{\ln r}. \end{array}$$

Let $0\leq l_{i}\leq L$ in this case, we find

(50) $$\begin{array}{cc} \frac{1}{n+(L-T)\ln r}\leq p_{i}\leq & \left\{\begin{array}{c} \frac{1}{n-T\ln r}\;\mathrm{if}\;\;\;n>T\ln r.\\ 1\;\;\;\;\;\;\;\;\;\;\mathrm{if}\;\;\;n\leq T\ln r. \end{array}\right. \end{array}$$

Generally, this constraint does not invariably hold, and therefore we usually do not have $\tilde{N}=n$.

For the quantification storage system as shown in $P_{3}$ in this section, if the maximum available average storage size satisfies n≤Tlnr, an arbitrary probability distribution will make Equation (50) hold, which means $\tilde{N}=n$. In this case, substituting Equation (47) in Equation (9), the RWRE can be expressed as

(51) $$\begin{array}{c} D_{r}\left(x,W\right)=\frac{\sum_{i=1}^{n}p_{i}e^{\left(1-p_{i}\right)/p_{i}}r^{-l_{i}}}{\sum_{i=1}^{n}p_{i}e^{\left(1-p_{i}\right)/p_{i}}}=e^{n-1-L(P)}r^{-T}, \end{array}$$

where $L\left(P\right)=\ln\sum_{i=1}^{n}p_{i}e^{\left(1-p_{i}\right)/p_{i}}$, which is defined as the NMIM [23].

It is noted $D_{r}\left(x,W\right)=0$ as *T* approaches positive infinity. Since n≤Tlnr, we find $D_{r}\left(x,W\right)\leq r^{-1-L(P)}$. Furthermore, since that L(P)≥n−1 according to Reference [23], we obtain $D_{r}\left(x,W\right)\leq r^{-n}$. Let $D_{r}\left(x,W\right)\leq \delta$, we have

(52) $$\begin{array}{c} T\geq \frac{n-1-L(P)-\ln\delta }{\ln r}. \end{array}$$

Obviously, for a given RWRE, the minimum average required storage size for the quantification storage system decreases with increasing of L(P). That is to say, the data with large NMIM will get a large compression ratio. In fact, the NMIM in the typical small-probability event scenarios is generally large according to Reference [23]. Thus, this compression strategy is effective in the typical small-probability event scenarios.

Furthermore, due to Reference [23], $L\left(P\right)\approx \ln p_{\alpha_{1}}e^{\frac{1-p_{\alpha_{1}}}{p_{\alpha_{1}}}}$ when $p_{\alpha_{1}}$ is small. Hence, for small $p_{\alpha_{1}}$, the RWRE in this case can be reduced to

(53) $$\begin{array}{c} D_{r}\left(x,W\right)\approx \frac{e^{n-1/p_{\alpha_{1}}}}{p_{\alpha_{1}}}r^{-T}. \end{array}$$

It is easy to check that $D_{r}\left(x,W\right)$ increases as $p_{\alpha_{1}}$ increases in this case.

## 6. Numerical Results

We now present numerical results to validate the developed theoretical results in this paper. In this section, we assume all the data is digital, and the exponential distortion measure $D_{f}$ in Equation (4) is adopted. Furthermore, the relative weighted reconstruction error (RWRE) in Equation (5) is used to characterize the change of total data value before and after the lossy compression based on data value, which represents the total cost of this compression.

### 6.1. Success Rate of Compressed Storage in General Storage System

This part presents the success rate of compressed storage in the general storage system to show the effectiveness of our method, and it considers the following scenario of data storage.

**Figure 3:**
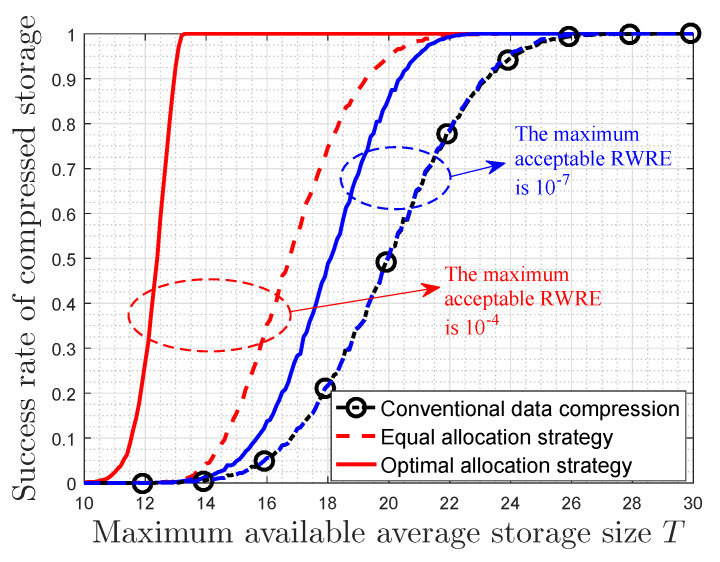
The success rate of compressed storage versus the maximum available average storage size.

There are eight categories of data, and the probability distribution of the data category randomly generates in each storing. Moreover, each category of data gets a randomly generated data value, which is in the interval (0,100). After the lossless conventional data compression, where the data value is assumed to be unchanged, the storage size of each data is a randomly generated number between 10 and 30. The maximum available average storage size *T* is also varying from 10 to 30 bits. It is considered as a successful data compression when the compressed storage size is not larger than the maximum available storage size. However, when the amount of data to be stored is extremely big, the compressed storage size may still not be enough after the lossless conventional data compression. In this case, the optimal storage space allocation strategy in this paper can be used if a certain amount of data value is allowed to be lost. As a contrast, we also divide up the maximum available storage space equally among all categories of data on the basis of the lossless conventional data compression, which is presented as the equal allocation strategy in Figure 3. Assume that it can also be seen as a successful data compression if the RWRE in this process is less than or equal to the specified amount that can be acceptable by users. For each value of *T*, this numerical simulation is repeated 10,000 times. The success rate of compressed storage is given by $N_{s}$/10,000, where $N_{s}$ is the number of times the successful data compression happens in all the experiments.

Figure 3 shows the relationship between the success rate of compressed storage and the maximum available average storage size *T*. It is observed that the success rate of conventional data compression is almost one when the available storage size is large (T>26 bits). However, when the available storage size is not big (T<26 bits), the success rate of conventional data compression decreases with decreasing of the maximum available average storage size until it is zero. Furthermore, when a certain amount of data value is allowed to be lost, the success rate can be improved on the basis of the lossless conventional data compression for the same *T*. More important, the success rate of the optimal allocation strategy is the largest among these three considered compression strategies. For the same maximum available average storage size, the success rates of the optimal allocation strategy and the equal allocation strategy increase as the maximum acceptable RWRE increases. In fact, the success rate of equal allocation strategy is exceedingly close to that of conventional data compression when the maximum acceptable RWRE is small (e.g., $10^{-7}$). In general, if a small quantity of total data value is allowed to be lost, our optimal allocation strategy will further improve the performance of data compression on the basis of the lossless conventional data compression.

### 6.2. Optimal Storage Size Based on Message Importance Measure in Ideal Storage System

We illustrate the characteristics of optimal storage size based on message importance measure (MIM) in an ideal storage system in this part by means of a broken line graph, which demonstrates the theoretical analyses in Section 4.2. For ease of illustrating, we ignore rounding and the optimal storage size of the *i*-th class is given by $l_{i}$ in Equation (19).

**Figure 4:**
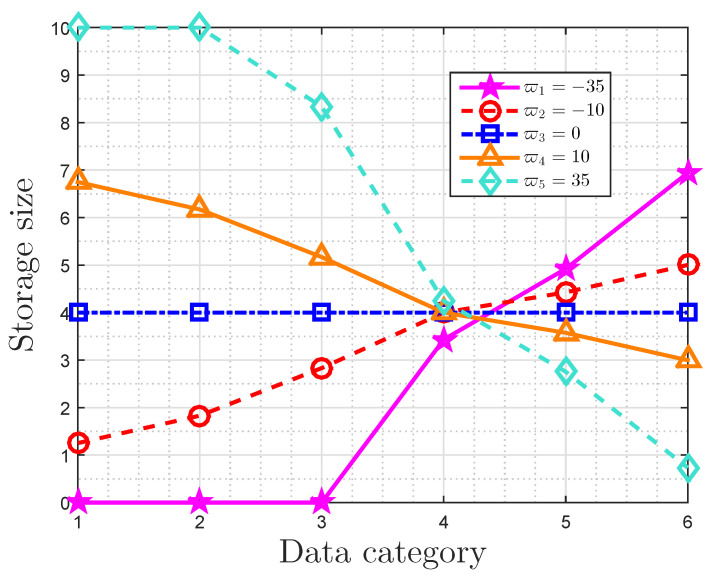
Broken line graph of optimal storage size with the probability distribution (0.03,0.07,0.1395,0.2205,0.25,0.29), for a given maximum available average storage size T=4 and original storage size L=10.

The broken line graph of the optimal storage size is shown in Figure 4, when the probability distribution is P=(0.03,0.07,0.1395,0.2205,0.25,0.29). In fact, $0.2205\approx \gamma_{P}$ and 1/n≈0.167. The maximum available average storage size *T* is 4 bits, and the original storage size of each data is 10 bits. The importance coefficients are given by $\varpi_{1}=-35,\varpi_{2}=-10,\varpi_{3}=0,\varpi_{4}=10,\varpi_{5}=35$, respectively. Some observations can be obtained. When ϖ>0, the optimal storage size of the *i*-th class decreases with the increasing of its probability. On the contrary, the optimal storage size of the *i*-th class increases as its probability increases when ϖ<0. In addition, the optimal storage size is invariably equal to *T* (T=4) when ϖ=0. Furthermore, $l_{i}$ increases as ϖ increases for i=1,2,3, and it decreases with ϖ for i=5,6. For importance coefficients with small absolute values ($\varpi_{2},\varpi_{3},\varpi_{4}$), $0<l_{i}<L$ holds for i=1,2,…,6, and $l_{4}$ is extremely close to *T* (T=4).

### 6.3. The Property of the RWRE Based on MIM in Ideal Storage System

Then we focus on the properties of the RWRE. In this part, we will give several numerical results as quintessential examples to validate our theoretical founds in Section 4.3. Without loss of generality, let the original storage size of each data be 16 bits, and the maximum available average storage size *T* is varying from 0 to 8 bits. Although any range of *T* can be used, we choose this range to make the results more clear. Moreover, the normalized MIM is adopted to describe the data value that represents the subjective assessment of users.

**Figure 5:**
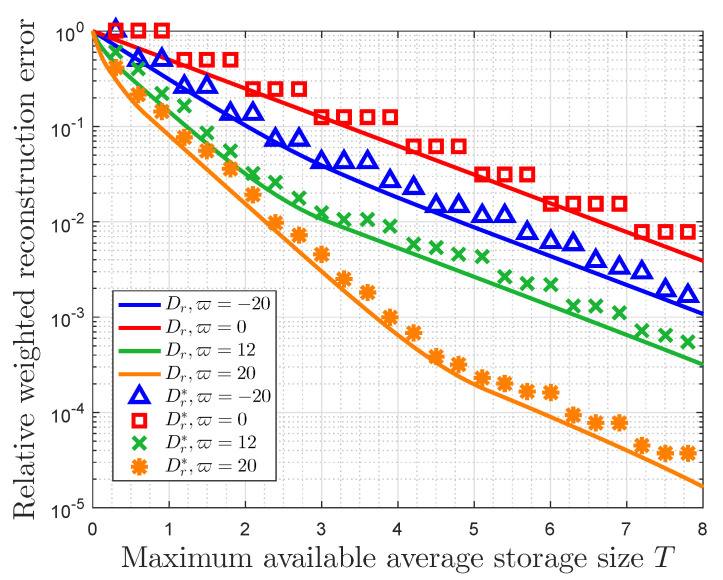
Relative weighted reconstruction error (RWRE) $D_{r}\left(x,\varpi \right)$ versus maximum available average storage size *T* with the probability distribution (0.031,0.052,0.127,0.208,0.582) in the case of the value of importance coefficient ϖ=−20,0,−12,20. $D_{r}$ is acquired by substituting Equation (19) in Equation (38), while $D_{r}^{*}$ is obtained by substituting Equation (22) in Equation (38).

**Figure 6:**
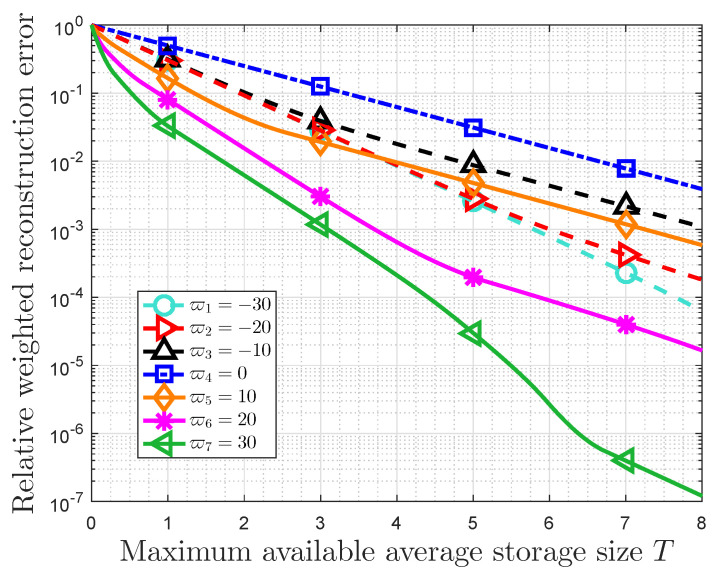
RWRE $D_{r}\left(x,\varpi \right)$ versus maximum available average storage size *T* with the probability distribution (0.031,0.052,0.127,0.208,0.582) in the case of the value of importance coefficient ϖ=−30,−20,−10,0,10,20,30.

Figure 5 and Figure 6 both present the relationship between the RWRE and the maximum available average storage size with the probability distribution (0.031,0.052,0.127,0.208,0.582). In fact, the compression ratio is given by T/L in this case, and the RWRE represents the total cost, which measures the compression distortion from the viewpoint of data value. Therefore, these two figures essentially show the trade-off between the compression ratio and the total compressed storage cost.

Figure 5 focuses on the error of RWRE by rounding number with different values of the importance coefficient ϖ (ϖ=−20,0,−12,20). In Figure 5, the RWRE $D_{r}$ is acquired by substituting Equation (19) in Equation (38), while the RWRE $D_{r}^{*}$ is obtained by substituting Equation (22) in Equation (38). In this figure, $D_{r}^{*}$ has a tiered descent as the available average storage size increases, while $D_{r}$ monotonically decreases with increasing in the available average storage size. Figure 5 also shows that $D_{r}$ is always less than or equal to $D_{r}^{*}$ and they are very close to each other for the same importance coefficient, which means that $D_{r}$ can be used as the lower bound of $D_{r}^{*}$ to reflect the characteristics of $D_{r}^{*}$.

Furthermore, some other observations can be obtained in Figure 6. For the same *T*, the RWRE increases as ϖ increases when ϖ<0, while the RWRE decreases with increasing of ϖ when ϖ>0. In addition, the RWRE is the largest when ϖ=0. These results prove the validity of Theorem 4. It is also observed that the RWRE always decreases with increasing of *T* for given ϖ. Furthermore, for any importance coefficient, the RWRE will be 1 if available average storage size is zero. Generally, there is a trade-off between the RWRE and the available storage size, and the results in this paper propose an alternative lossy compression strategy based on message importance.

**Figure 7:**
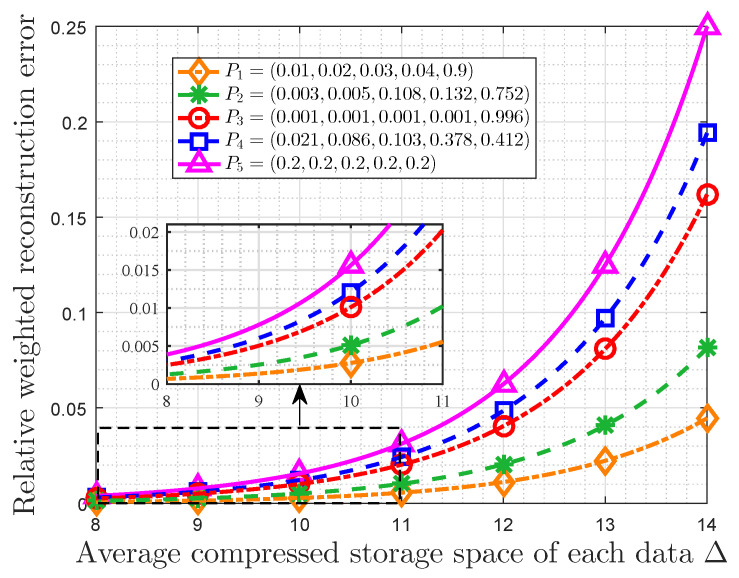
RWRE $D_{r}\left(x,\varpi \right)$ vs. average compressed storage size of each data Δ with importance coefficient ϖ=5.

Then let the importance coefficient ϖ be five and the maximum available average storage size *T* be varying from two to eight bits. Although any range of *T* can be used, we choose this range to make the results more clear. In addition, the original storage size is still 16 bits. Furthermore, the average compressed storage space of each data is given by Δ=L−T. In this case, Figure 7 shows that the relationship between the RWRE and the average compressed storage space of each data Δ for different probability distributions. In fact, it can also be seen as reflecting the relationship between the total compressed storage cost and the average saving storage size. The probability distributions and some auxiliary variables are listed in Table 2. In fact, we take these five probability distributions as examples, and $L\left(\varpi ,P\right)+\varpi e^{-H_{2}\left(P\right)}$ of them decreases monotonously. Obviously, all probability distributions satisfy $0\leq T+\varpi (\gamma_{p}-p_{i})/\ln r\leq L$. It is observed that the RWRE always increases with increasing of Δ for a given probability distribution. Some other observations are also obtained. For the same Δ, the RWRE of uniform distribution is the largest all the time. Furthermore, if the RWRE is required to be less than a specified value, which is exceedingly common in actual systems in order to make the difference between the raw data and the stored data accepted, the maximum average compressed storage size of each data will increase with increasing of $L\left(\varpi ,P\right)+\varpi e^{-H_{2}\left(P\right)}$. As an example, when the RWRE is required to be smaller than 0.01, the maximum average compressed storage size of $P_{1}$, $P_{2}$, $P_{3}$, $P_{4}$, $P_{5}$ of each data is 11.85, 10.97, 9.99, 9.73, 9.36, respectively. In particular, the maximum average compressed storage size of each data in a uniform distribution is the smallest, which suggests the data with a uniform distribution is incompressible from the perspective of optimal storage space allocation based on the data value.

### 6.4. The Property of the RWRE Based on Non-Parametric MIM in a Quantification Storage System

**Figure 8:**
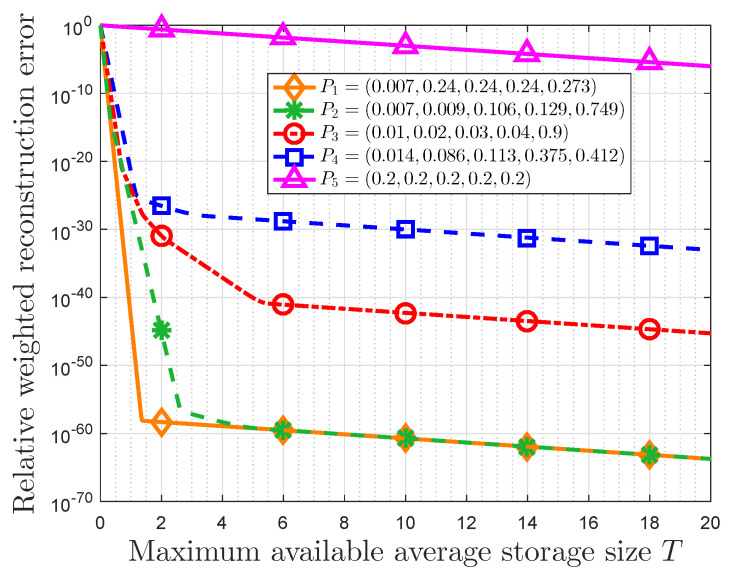
RWRE versus maximum available average storage size *T*.

Figure 8 presents the relationship between the RWRE and the maximum available average storage size *T* for different probability distributions in a quantification storage system, which proves the validity of theoretical results in Section 5. In this part, we use the normalized non-parametric message importance measure (NMIM) to characterize the data value that represents the subjective assessment of users. The probability distributions and some auxiliary variables are listed in Table 3.

Some observations can be obtained. First, the RWRE always decreases with the increasing of the maximum available average storage size for a given probability distribution, and there is a trade-off between the RWRE and the maximum available average storage size. When the maximum available average storage size is small (T<n/lnr), the RWRE decreases largely compared to the case where *T* is large. In addition, when the maximum available average storage size is large (T>n/lnr), the difference between these RWREs remains the same at the logarithmic Y-axis. In fact, according to Equation (51), this difference between two probabilities in this figure is the difference of NMIM divided by log10. As an example, the difference between $P_{1}$ and $P_{4}$ in this figure is 30, which satisfies this conclusion for the fact that $(L\left(P_{1}\right)-L\left(P_{4}\right))/\log10\approx 30$. Moreover, the RWRE in $P_{1}$ is very close to that in $P_{2}$, and the minimum probabilities in these two probability distributions are the same, i.e., $p_{\alpha_{1}}=0.007$. It suggests that the data with the same minimum probability will have the same compression performance no matter how the distribution changes, if the minimum probability is very small. In addition, it is also observed that the RWRE decreases as NMIM L(P) increases for the same *T*, which means this compression strategy is more effective in the large NMIM cases.

## 7. Conclusions

In this paper, we focused on the problem of lossy compression storage when the storage size is still not enough after conventional lossless data compression. By means of the message importance to characterize the data value, we define the weighted reconstruction error to describe the total cost in data storage. Based on it, we presented an optimal storage space allocation strategy for digital data from the perspective of data value by the exponential distortion measure, which pursues the least error with respect to the data value for restricted storage size. We gave the solutions by a kind of restrictive water-filling, which presented an alternative way to design an effective storage space allocation strategy. In fact, this optimal allocation strategy prefers to provide more storage size for crucial event classes in order to make the rational use of resources, which agrees with the individuals’ cognitive mechanism.

Then, we presented the properties of this strategy based on the message importance measure (MIM) detailedly. It is obtained that there is a trade-off between the relative weighted reconstruction error (RWRE) and available storage size. In fact, if a small quantity of loss of total data value is accepted by users, this strategy will further compress data based on the conventional methods of data compression. Moreover, the compression performance of this storage system improves as the absolute value of importance coefficient increases. This is due to the fact that a fraction of data can contain the overwhelming majority of useful information that exerts a tremendous fascination on users as the importance coefficient approaches negative/positive infinity, which suggests that the users’ interest is highly-concentrated. On the other hand, the probability distribution of event classes also has an effect on the compression results. When the useful information is only highly enriched in a small portion of raw data naturally from the viewpoint of users, such as the small-probability event scenarios, it is obvious that we can compress the data greatly with the aid of these characteristics of distribution. Furthermore, the properties of storage size and RWRE based on non-parametric MIM were also discussed. In fact, the RWRE in the data with a uniform distribution was invariably the largest in any case. Therefore, this paper harbors the idea that the data with uniform information distribution is incompressible from the perspective of optimal storage size allocation based on data value, which is consistent with the well known conclusion in information theory in a sense.

Proposing a more general distortion measure between the raw data and the compressed data, which no longer only applies to digital data, and using it to acquire the high-efficiency lossy data compression systems from the perspective of message importance are of our future interests. In addition, we are also interested in using this optimal storage space allocation strategy in a real application with a real data stream in the future.

## Figures and Tables

**Table 1:** Notations.

Notation	Description
$x=x_{1},x_{2},\ldots ,x_{k},\ldots ,x_{K}$	The sequence of raw data
$\hat{x}={\hat{x}}_{1},{\hat{x}}_{2},\ldots ,{\hat{x}}_{k},\ldots ,{\hat{x}}_{K}$	The sequence of compressed data
$S_{x}$	The storage size of *x*
$D_{f}\left(S_{x1},S_{x2}\right)$	The distortion measure function between $S_{x1}$ and $S_{x2}$ in data reconstruction
*n*	The number of event classes
$\left\{a_{1},a_{2},\ldots ,a_{n}\right\}$	The alphabet of raw data
$\left\{{\hat{a}}_{1},{\hat{a}}_{2},\ldots ,{\hat{a}}_{n}\right\}$	The alphabet of compressed data
$W=\{W_{1},W_{2},\ldots ,W_{n}\}$	The error cost for the reconstructed data
$P=\{p_{1},p_{2},\ldots ,p_{n}\}$	The probability distribution of data class
D(x,W)	The weighted reconstruction error
$D_{r}\left(x,W\right),D_{r}\left(W,L,l\right)$	The relative weighted reconstruction error
$L=L_{1},L_{2},\ldots ,L_{n}$	The storage size of raw data
$l=l_{1},l_{2},\ldots ,l_{n}$	The storage size of compressed data
$l_{i}^{*}$	The round optimal storage size of the data belonging to the *i*-th class
*T*	The maximum available average storage size
ϖ	The importance coefficient
$\gamma_{p}$	$\gamma_{p}=\sum_{i=1}^{n}p_{i}^{2}$
$\alpha_{1}$, $\alpha_{2}$	$\alpha_{1}=\arg\min_{i}p_{i}$ and $\alpha_{2}=\arg\max_{i}p_{i}$
L(ϖ,p)	The message importance measure, which is given by $L\left(\varpi ,p\right)=\ln\sum_{i=1}^{n}p_{i}e^{\varpi (1-p_{i})}$
Δ	The average compressed storage size of each data, which is given by Δ=L−T
$\Delta^{*}\left(\delta \right)$	The maximum available Δ for the given supremum of the RWRE δ
L(P)	The non-parametric message importance measure, which is given by $L\left(P\right)=\ln\sum_{i=1}^{n}p_{i}e^{\left(1-p_{i}\right)/p_{i}}$

**Table 2:** The auxiliary variables in ideal storage system.

Variable	Probability Distribution	$\varpi (\gamma_{p}-p_{\alpha_{1}})/\ln r$	$\varpi (\gamma_{p}-p_{\alpha_{2}})/\ln r$	$L\left(\varpi ,P\right)+\varpi e^{-H_{2}\left(P\right)}$
$P_{1}$	(0.01,0.02,0.03,0.04,0.9)	5.7924	−0.6276	6.7234
$P_{2}$	(0.003,0.007,0.108,0.132,0.752)	4.2679	−1.1350	6.1305
$P_{3}$	(0.001,0.001,0.001,0.001,0.996)	7.1487	−0.0287	5.4344
$P_{4}$	(0.021,0.086,0.103,0.378,0.412)	2.2367	−0.5838	5.2530
$P_{5}$	(0.2,0.2,0.2,0.2,0.2)	0	0	5

**Table 3:** The auxiliary variables in the quantification storage system.

Variable	Probability Distribution	$p_{\alpha_{1}}$	L(P)
$P_{1}$	(0.007,0.24,0.24,0.24,0.273)	0.007	136.8953
$P_{2}$	(0.007,0.009,0.106,0.129,0.749)	0.007	136.8953
$P_{3}$	(0.01,0.02,0.03,0.04,0.9)	0.01	94.3948
$P_{4}$	(0.014,0.086,0.113,0.375,0.412)	0.014	66.1599
$P_{5}$	(0.2,0.2,0.2,0.2,0.2)	0.2	4.0000

## Abbreviations

The following abbreviations are used in this manuscript:

IoT	Internet of things
MIM	message importance measure
RWRE	relative weighted reconstruction error
NMIM	non-parametric message importance measure

## Appendix A. Proof of Theorem 2

In fact, Equation (25) can be rewritten as

(A1) $$\begin{array}{cc} l_{i}= & \left\{\begin{array}{c} \;\;0\;\;\;\;\;\;\;\;\;\;\;\;\;\mathrm{if}\;\;W_{i}<e^{-\beta \ln r}.\\ \beta -\frac{-\ln W_{i}}{\ln r}\;\;\;\;\;\;\;\mathrm{if}\;\;e^{-\beta \ln r}\leq W_{i}\leq e^{(L-\beta )\ln r}.\\ \;\;L\;\;\;\;\;\;\;\;\;\;\;\;\;\mathrm{if}\;\;W_{i}>e^{(L-\beta )\ln r}. \end{array}\right. \end{array}$$

When $W_{i}>W_{j}$, we have

(A2) $$\begin{array}{cc} l_{i}-l_{j}= & \left\{\begin{array}{c} 0\;\;\;\;\;\;\;\;\;\;\mathrm{if}\;\;\;p_{i}>e^{(L-\beta )\ln r},\;\;\;p_{j}>e^{(L-\beta )\ln r}.\\ L-\beta -\frac{\ln W_{j}}{\ln r}\;\;\;\;\;\mathrm{if}\;\;\;p_{i}>e^{(L-\beta )\ln r},\;\;\;e^{-\beta \ln r}\leq p_{j}\leq e^{(L-\beta )\ln r}.\\ L\;\;\;\;\;\;\;\;\;\;\;\;\;\;\mathrm{if}\;\;\;p_{i}>e^{(L-\beta )\ln r},\;\;\;p_{j}<e^{-\beta \ln r}.\\ \frac{\ln W_{i}-\ln W_{j}}{\ln r}\;\;\;\;\;\;\mathrm{if}\;\;\;e^{-\beta \ln r}\leq p_{i}\leq e^{(L-\beta )\ln r},\;\;\;e^{-\beta \ln r}\leq p_{j}\leq e^{(L-\beta )\ln r}.\\ \beta -\frac{-\ln W_{i}}{\ln r}\;\;\;\;\;\;\mathrm{if}\;\;\;e^{-\beta \ln r}\leq p_{i}\leq e^{(L-\beta )\ln r},\;\;\;p_{j}<e^{-\beta \ln r}.\\ 0\;\;\;\;\;\;\;\;\;\;\mathrm{if}\;\;\;p_{i}<e^{-\beta \ln r},\;\;\;p_{j}<e^{-\beta \ln r}. \end{array}\right. \end{array}$$

Due to Equation (8b), we obtain that $0\leq \beta -\frac{-\ln W_{i}}{\ln r}\leq L$, and therefore $L-\beta -\frac{\ln W_{j}}{\ln r}\geq 0$. Furthermore, it is easy to check that $\frac{\ln W_{i}-\ln W_{j}}{\ln r}$ since that $W_{i}>W_{j}$. Thus, $l_{i}-l_{j}\geq 0$ if $W_{i}>W_{j}$ for ∀i,j∈{1,2,…,n}. The proof is completed.

## Appendix B. Proof of Lemma 1

(1) For $\gamma_{p}$, it is noted that

(A3) $$\begin{array}{c} \sum_{i=1}^{n}p_{i}^{2}=\frac{1}{n}\left(\sum_{i=1}^{n}p_{i}^{2}\sum_{i=1}^{n}1^{2}\right)\geq \frac{1}{n}{\left(\sum_{i=1}^{n}p_{i}\right)}^{2}=\frac{1}{n}, \end{array}$$

where the equality holds only if $\left(p_{1},p_{2},\ldots ,p_{n}\right)$ is a uniform distribution. Moreover,

(A4) $$\begin{array}{c} \sum_{i=1}^{n}p_{i}^{2}\leq \sum_{i=1}^{n}p_{i}=1, \end{array}$$

where the equality holds only if there is only $p_{t}=1$ (t∈{1,2,…,n}) and $p_{k}=0$ for k≠t.

(2) For $\gamma_{p}-p_{i}$, we have $\sum_{i=1}^{n}p_{i}^{2}-p_{i}\leq \sum_{i=1}^{n}p_{i}^{2}\leq 1$. We have equality if and only if $p_{t}=1$ and $p_{i}=0$ for i≠t. Therefore, we only need to check $\sum_{i=1}^{n}p_{i}^{2}-p_{i}\geq -1/4$.

First, if n=1, we obtain $\sum_{i=1}^{n}p_{i}^{2}-p_{i}=0$.

Second, if n=2, we obtain $\sum_{i=1}^{n}p_{i}^{2}-p_{i}=2{\left(p_{1}-3/4\right)}^{2}-1/8$. It is easy to check that $\sum_{i=1}^{n}p_{i}^{2}-p_{i}\geq -1/8$.

Third, if n>3, we use the method of Lagrange multipliers. Let

(A5) $$\begin{array}{c} J\left(p\right)=\sum_{j=1}^{n}p_{j}^{2}-p_{i}-\lambda \left(\sum_{j=1}^{n}p_{j}-1\right). \end{array}$$

Setting the derivative to 0, we obtain

(A6) $$\begin{array}{cc} 2p_{j}^{*}-\lambda & =0\;\;\;\mathrm{for}\;j\neq i \end{array}$$

(A6a) $$\begin{array}{cc} 2p_{j}^{*}-1-\lambda & =0\;\;\;\mathrm{for}\;j=i. \end{array}$$

Substituting $p_{j}^{*}$ in the constraint $\sum_{j=1}^{n}p_{j}^{*}=1$, we have

(A7) $$\begin{array}{c} \frac{\lambda (n-1)}{2}+\frac{\lambda +1}{2}=1. \end{array}$$

Hence, we find λ=1/n and

(A8) $$\begin{array}{cc} p_{j}^{*}= & \left\{\begin{array}{c} \frac{n+1}{2n}\;\;\mathrm{if}\;\;j=i,\\ \frac{1}{2n}\;\;\;\;\mathrm{if}\;\;j\neq i. \end{array}\right. \end{array}$$

In this case, we get

(A9) $$\begin{array}{c} \sum_{j=1}^{n}p_{j}^{2}-p_{i}=\frac{n-1}{4n^{2}}+\frac{{\left(n+1\right)}^{2}}{4n^{2}}-\frac{n+1}{2n}=\frac{-n^{2}+n}{4n^{2}}\geq -\frac{1}{4}. \end{array}$$

Thus, Lemma 1 is proved.

## Appendix C. Proof of Theorem 3

(1) First, let $p_{i}<p_{j}$ when ϖ>0. It is noted that

(A10) $$\begin{array}{c} W_{i}=\frac{e^{\varpi (1-p_{i})}}{\sum_{k=1}^{n}e^{\varpi (1-p_{k})}}>\frac{e^{\varpi (1-p_{j})}}{\sum_{k=1}^{n}e^{\varpi (1-p_{k})}}=W_{j}. \end{array}$$

Therefore, we find $l_{i}\geq l_{j}$ since that $W_{i}>W_{j}$, due to Theorem 2.

(2) Second, let $p_{i}<p_{j}$ when ϖ<0. It is noted that

(A11) $$\begin{array}{c} W_{i}=\frac{e^{\varpi (1-p_{i})}}{\sum_{k=1}^{n}e^{\varpi (1-p_{k})}}<\frac{e^{\varpi (1-p_{j})}}{\sum_{k=1}^{n}e^{\varpi (1-p_{k})}}=W_{j}. \end{array}$$

Therefore, we find $l_{i}\leq l_{j}$ since that $W_{i}<W_{j}$, due to Theorem 2. The proof is completed.

## Appendix D. Proof of Theorem 4

We define an auxiliary function as

(A12) $$\begin{array}{c} f\left(\varpi \right)=\frac{\sum_{i=1}p_{i}e^{\varpi (1-p_{i})}r^{-l_{i}}}{\sum_{j=1}^{n}p_{j}e^{\varpi (1-p_{j})}}. \end{array}$$

According to Equation (38), it is noted that the the monotonicity of $D_{r}\left(x,\varpi \right)$ with respect to ϖ is the same with that of f(ϖ).

Without loss of generality, let $l_{i}$ of $p_{i}$ be

(A13) $$\begin{array}{cc} l_{i}= & \left\{\begin{array}{c} \;\;\;L\;\;\;\;\;\;\;\;\;\;\;\;\;\;\;\;\;\mathrm{if}\;\;i=1,2,\ldots ,t_{1},\\ \frac{\ln\left(\ln r\right)+\ln W_{i}-\ln\lambda }{\ln r}\;\mathrm{if}\;\;i=t_{1}+1,\ldots ,t_{2},\\ \;\;\;0\;\;\;\;\;\;\;\;\;\;\;\;\;\;\;\;\;\;\mathrm{if}\;\;i=t_{2}+1,t_{2}+2,\ldots ,n, \end{array}\right. \end{array}$$

where λ is given by Equation (20) where $\left\{T_{j},j=1,\ldots ,{\tilde{N}}_{L}\right\}=\left\{1,2,\ldots ,t_{1}\right\}$ and $\left\{I_{j},j=1,\ldots ,\tilde{N}\right\}=\left\{t_{1}+1,\ldots ,t_{2}\right\}$.

The derivative of $l_{i}$ with respect to ϖ is given by

(A14) $$\begin{array}{cc} l_{i}^{\prime }= & \left\{\begin{array}{c} \frac{\sum_{k=t_{1}+1}^{t_{2}}p_{k}\left(p_{k}-p_{i}\right)}{\ln r(\sum_{k=t_{1}+1}^{t_{2}}p_{k})}\;\;\mathrm{if}\;\;\;i=t_{1}+1,\ldots ,t_{2}.\\ \;\;0\;\;\;\;\;\;\;\;\;\;\mathrm{else}. \end{array}\right. \end{array}$$

Hence,

(A15) $$\begin{array}{cc} f^{\prime }\left(\varpi \right) & =\frac{\sum_{i}\sum_{j}p_{i}p_{j}e^{\varpi (2-p_{i}-p_{j})}r^{-l_{i}}\left(p_{j}-p_{i}-l_{i}^{\prime }\ln r\right)}{{\left(\sum_{j}p_{j}e^{\varpi (1-p_{j})}\right)}^{2}}=\frac{F_{1}+F_{2}}{{\left(\sum_{j}p_{j}e^{\varpi (1-p_{j})}\right)}^{2}}, \end{array}$$

where $F_{1}=\sum_{i}\sum_{j}p_{i}p_{j}e^{\varpi (2-p_{i}-p_{j})}r^{-l_{i}}\left(p_{j}-p_{i}\right)$ and $F_{2}=\sum_{i}\sum_{j}p_{i}p_{j}e^{\varpi (2-p_{i}-p_{j})}r^{-l_{i}}\left(-l_{i}^{\prime }\ln r\right)$.

(1) When ϖ>0, we have

(A16) $$\begin{array}{cc} F_{1} & =\sum_{p_{j}<p_{i}}p_{i}p_{j}e^{\varpi (2-p_{i}-p_{j})}r^{-l_{i}}\left(p_{j}-p_{i}\right)+\sum_{p_{j}>p_{i}}p_{i}p_{j}e^{\varpi (2-p_{i}-p_{j})}r^{-l_{i}}\left(p_{j}-p_{i}\right) \end{array}$$

(A16a) $$\begin{array}{c} \leq \sum_{p_{j}<p_{i}}p_{i}p_{j}e^{\varpi (2-p_{i}-p_{j})}r^{-l_{j}}\left(p_{j}-p_{i}\right)+\sum_{p_{j}>p_{i}}p_{i}p_{j}e^{\varpi (2-p_{i}-p_{j})}r^{-l_{i}}\left(p_{j}-p_{i}\right) \end{array}$$

(A16b) $$\begin{array}{c} =\sum_{p_{j}<p_{i}}p_{i}p_{j}e^{\varpi (2-p_{i}-p_{j})}r^{-l_{j}}\left(p_{j}-p_{i}\right)+\sum_{p_{i}>p_{j}}p_{i}p_{j}e^{\varpi (2-p_{i}-p_{j})}r^{-l_{j}}\left(p_{i}-p_{j}\right) \end{array}$$

(A16c) $$\begin{array}{c} =\sum_{p_{j}<p_{i}}p_{i}p_{j}e^{\varpi (2-p_{i}-p_{j})}r^{-l_{j}}\left(p_{j}-p_{i}+p_{i}-p_{j}\right) \end{array}$$

(A16d) $$\begin{array}{c} =0. \end{array}$$

In fact, if $p_{i}>p_{j}$, then we will have $l_{i}\leq l_{j}$ due to Theorem 3. Thus, $r^{-l_{i}}\left(p_{j}-p_{i}\right)\leq r^{-l_{j}}\left(p_{j}-p_{i}\right)$ in this case. With taking $p_{i}p_{j}e^{\varpi (2-p_{i}-p_{j})}\geq 0$ into account, we have Equation (A16a). Furthermore, Equation (A16b) is obtained by exchanging the notation of subscript in the second item.

For $t_{1}<i\leq t_{2}$ and 1≤j≤n, we have

(A17) $$\begin{array}{cc} F_{2} & =\sum_{i=t_{1}+1}^{t_{2}}\sum_{j=1}^{n}p_{i}p_{j}e^{\varpi (2-p_{i}-p_{j})}r^{-l_{i}}\left(-l_{i}^{\prime }\ln r\right) \end{array}$$

(A17a) $$\begin{array}{c} =\sum_{i=t_{1}+1}^{t_{2}}\sum_{j=1}^{n}p_{i}p_{j}e^{\varpi (1-p_{j})-\ln\ln r+\ln\lambda }\left(-l_{i}^{\prime }\ln r\right) \end{array}$$

(A17b) $$\begin{array}{c} =\sum_{j=1}^{n}\left(p_{j}B_{j}\left(\sum_{i=t_{1}+1}^{t_{2}}p_{i}\left(-l_{i}^{\prime }\ln r\right)\right)\right) \end{array}$$

(A17c) $$\begin{array}{c} =\sum_{j=1}^{n}\left(p_{j}B_{j}\left(\frac{\sum_{i=t_{1}+1}^{t_{2}}p_{i}^{2}\sum_{k=t_{1}+1}^{t_{2}}p_{k}-\sum_{k=t_{1}+1}^{t_{2}}p_{k}^{2}\sum_{i=t_{1}+1}^{t_{2}}p_{i}}{\sum_{k=t_{1}+1}^{t_{2}}p_{k}}\right)\right) \end{array}$$

(A17d) $$\begin{array}{c} =0, \end{array}$$

where $B_{j}=\exp\{\varpi (1-p_{j})-\ln\ln r+\ln\lambda \}$.

Based on the discussions above, we have

(A18) $$\begin{array}{cc} f^{\prime }\left(\varpi \right) & =\frac{F_{1}+F_{2}}{{\left(\sum_{i=1}^{n}p_{i}e^{\varpi (1-p_{i})}\right)}^{2}}\leq 0. \end{array}$$

Since that $f^{\prime }\left(\varpi \right)\leq 0$ when ϖ>0, $D_{r}\left(x,\varpi \right)$ is monotonically decreasing with ϖ in (0,+∞).

(2) Similarly, when ϖ<0, if $0<p_{j}<p_{i}$, then we will have $l_{i}>l_{j}$ due to Theorem 3. Thus, $r^{-l_{i}}\left(p_{j}-p_{i}\right)\geq r^{-l_{j}}\left(p_{j}-p_{i}\right)$ in this case. With taking $p_{i}p_{j}e^{\varpi (2-p_{i}-p_{j})}\geq 0$ into account, we have

(A19) $$\begin{array}{cc} F_{1} & \geq \sum_{p_{j}<p_{i}}p_{i}p_{j}e^{\varpi (2-p_{i}-p_{j})}r^{-l_{j}}\left(p_{j}-p_{i}\right)+\sum_{p_{j}>p_{i}}p_{i}p_{j}e^{\varpi (2-p_{i}-p_{j})}r^{-l_{i}}\left(p_{j}-p_{i}\right) \end{array}$$

(A19a) $$\begin{array}{c} =\sum_{p_{j}<p_{i}}p_{i}p_{j}e^{\varpi (2-p_{i}-p_{j})}r^{-l_{j}}\left(p_{j}-p_{i}\right)+\sum_{p_{i}>p_{j}}p_{i}p_{j}e^{\varpi (2-p_{i}-p_{j})}r^{-l_{j}}\left(p_{i}-p_{j}\right) \end{array}$$

(A19b) $$\begin{array}{c} =\sum_{p_{j}<p_{i}}p_{i}p_{j}e^{\varpi (2-p_{i}-p_{j})}r^{-l_{j}}\left(p_{j}-p_{i}+p_{i}-p_{j}\right) \end{array}$$

(A19c) $$\begin{array}{c} =0, \end{array}$$

where Equation (A19a) is obtained by exchanging the notation of subscript in the second item.

In addition, $F_{2}$ is still given by Equation (A17), and $F_{2}=0$. As a result, $f^{\prime }\left(\varpi \right)\geq 0$ when ϖ<0. Therefore $D_{r}\left(x,\varpi \right)$ is monotonically increasing with ϖ in (−∞,0).

(3) When ϖ=0, the storage size $l_{i}$ for i=1,2,…,n will be all equal to *T*, and therefore $D_{r}\left(x,0\right)=\left(r^{L-T}-1\right)/\left(r^{L}-1\right)$. Based on the discussion in (1) and (2), we obtain $D_{r}\left(x,\varpi \right)\leq D_{r}\left(x,0\right)$. The proof is completed.
